# Variations in the Chemical Composition of Essential Oils in Native Populations of Korean Thyme, *Thymus quinquecostatus* Celak.

**DOI:** 10.3390/molecules27217203

**Published:** 2022-10-24

**Authors:** Minju Kim, Kandhasamy Sowndhararajan, Ponnuvel Deepa, Songmun Kim

**Affiliations:** 1 School of Natural Resources and Environmental Science, Kangwon National University, Chuncheon 24341, Korea; 2 Department of Botany, Kongunadu Arts and Science College, Coimbatore 641029, Tamil Nadu, India

**Keywords:** *Thymus quinquecostatus*, thyme, chemotypes, essential oil, cluster analysis

## Abstract

The genus *Thymus* (Lamiaceae) contains numerous medicinally important species. Among them, *Thymus quinquecostatus* Celak. has been extensively utilized as a traditional medicine and a food flavoring agent in the Korean peninsula, owing to its unique aroma. In particular, *T. quinquecostatus* has been used for the treatment of gastroenteritis, inflammation, stomach problems, liver disease, arthritis, arteriosclerosis, and menstrual problems. This study aimed to investigate the chemical diversity of essential oils among 103 Korean native populations of *T. quinquecostatus*. For this purpose, seedlings of *T. quinquecostatus* populations were purchased from different regions in the Korean Peninsula, and seedlings were grown in the experimental field under the same environmental conditions. The chemical compositions of steam-distilled essential oils were determined using GC-MS. In total, 212 components were identified from 103 populations of *T. quinquecostatus*. Furthermore, principal component analysis (PCA) was performed in order to understand variations in the essential oil compositions among 103 Korean native populations of *T. quinquecostatus*. According to the essential oil compositions, 30 components were selected for PCA. Based on the most abundant essential oil components, four chemotypes were identified in *T. quinquecostatus* populations. PCA and cluster analyses revealed that 103 individuals of *T. quinquecostatus* could be classified into four clusters, such as thymol, geraniol, geranyl acetate, and linalool. Furthermore, dendrogram construction demonstrated that geraniol and geranyl acetate, as well as linalool and thymol groups, were closely related. This study suggested the significant chemical polymorphism of essential oils in local populations of *T. quinquecostatus* in Korea. It could be concluded that the intraspecific variations in the essential oil compositions may be associated with genetic diversity among the individuals.

## 1. Introduction

Essential oils exhibit various biological properties, due to the presence of thousands of low-molecular-weight volatile components in them, primarily terpenes and their oxygenated derivatives, such as alcohols, aldehydes, esters, and phenols [1]. Essential oils isolated from *Thymus* species are well known for their potential use in food industries as flavoring agents, due to their unique fragrances. The genus *Thymus* (Lamiaceae) contains about 300 species of medicinally important aromatic plants [2]. In traditional systems of medicine, infusions and decoctions obtained from the leaves and flowers of *Thymus* species have been utilized in the treatments of several diseases, including complications in digestion, in addition to circulatory, genital, nervous, urinary, skin, and respiratory conditions [3]. It has been shown that essential oils and compounds isolated from different *Thymus* species exhibited strong antioxidant, antimicrobial, anti-tumoral, insecticidal, anti-inflammatory, and neuroprotective properties [2,3,4]. Essential oils obtained from different *Thymus* species (Lamiaceae) mainly contain 35–55% thymol, followed by carvacrol, geraniol, and linalool. Thymol is widely used for flavor or as a fragrant material, in the preparation of herbal teas, and as antimicrobial and insecticidal agents [5].

Among the various species of *Thymus*, *T. quinquecostatus* Celak. is a medicinally important aromatic plant that is native to the Korean peninsula [5,6]. In Korea, there are two varieties of *T. quinquecostatus*, such as Bak-ri-hyang and Ulleungdo thyme (*T. quinquecostatus* var. *japonica*). In Korea, *T. quinquecostatus* has been traditionally used to treat diaphoretic, flatulence, and liver disorders, as well as stomachaches, menstrual problems, and coughs, in addition to being a flavoring agent. This plant has a high industrial value, due to its distinctive aroma [7]. Previous studies found that *T. quinquecostatus* has a lot of therapeutical potential. *T. quinquecostatus* extracts showed strong antioxidant and free radical scavenging potential [7,8], in addition to antimicrobial [9], anti-diabetic [10], anti-aging [11], hepatoprotective [12], anti-tumoral [13], and anti-inflammatory properties [14]. *T. quinquecostatus* extract effectively improved mitochondrial function and attenuated oxidative stress in lipopolysaccharide-induced RAW 264.7 macrophages [14]. A recent study reported that the polyphenol-rich extract of *T. quinquecostatus* ameliorated cerebral ischemia-reperfusion injuries in rats [15].

Essential oil composition is a significant quality parameter for the commercial applications of *T. quinquecostatus* species. Recently, several studies reported that the same plant species collected from different locations exhibited different essential oil compositions, mostly in the yield and concentrations of major components in the essential oils [16,17,18]. Previously, some studies reported on the chemical composition of *T. quinquecostatus* essential oils, and their compositions varied significantly according to different geographical origins [19,20,21,22,23]. The essential oil compositions of *T. quinquecostatus* cultivars that were collected from three mountains in Korea showed three different chemotypes, such as geraniol, thymol, and linalool [5]. The yield, chemical components, and biological activities of essential oils are severely influenced by various factors, including environmental conditions, genotype, growth stage, extraction technique, etc. [24,25]. Principal component analysis (PCA) is extensively employed to understand the relationships and similarities between, as well as within species, according to the major components in the essential oils. PCA analysis provides insight into the distribution of essential oil components in plant populations [4,26,27].

There are no detailed studies on the diversity in the composition of essential oils within Korean populations of *T. quinquecostatus*. Hence, the present study aimed to investigate the chemical diversity in the essential oils of *T. quinquecostatus* populations collected from different regions in the Korean peninsula via GC-MS analysis. Furthermore, PCA was performed, in order to identify intraspecific variations in *T. quinquecostatus* populations.

## 2. Results

### 2.1. The Yield and Color of T. quinquecostatus Essential Oils

A total of 107 (4 seedlings decayed due to being frozen) Korean native populations of *T. quinquecostatus* were selected in the present study (Table 1). Seedlings collected from 103 individuals were planted under the same environmental conditions, cultivated during the flowering stage, and used for the isolation of essential oils. The average yield of essential oils obtained from the aerial parts of *T. quinquecostatus* populations was 0.40% (*v*/*w*). The highest yield was obtained from the sample site T64 (0.8%) (Gwacheon-si, Gyeonggi-do) (Table 1). The color of extracted essential oil was classified into red, orange, dark yellow, yellow, pale yellow, transparent yellow, and lemon.

### 2.2. Essential Oil Compositions of T. quinquecostatus Populations

**Figure 1:**
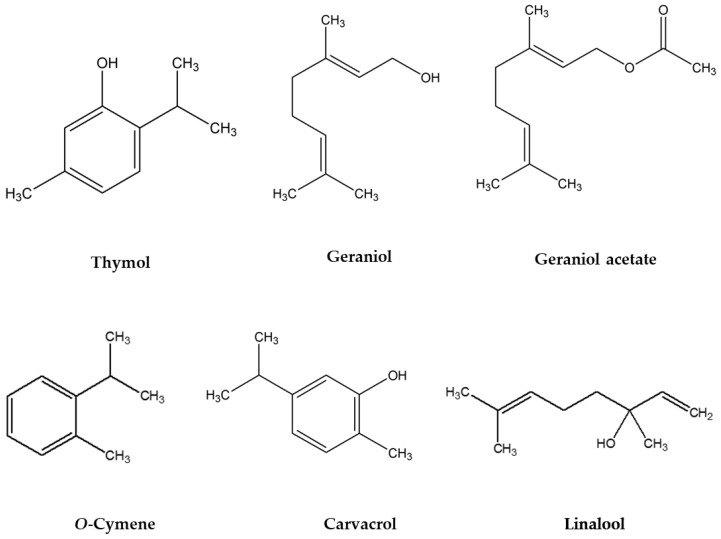
Chemical structures of different chemotypes identified in the essential oils of *T. quinquecostatus* populations.

Based on the GC-MS analyses, 212 different compounds were detected in the essential oils of 103 *T. quinquecostatus* individuals. All of the essential oil samples, qualitatively and quantitatively, exhibited different chemical compositions. Thymol, geraniol, geranyl acetate, and linalool were the predominant components in essential oils (Supplementary Table S1). Among them, 30 components were detected in most of the essential oils, and these components were used for further PCA studies. Table 2 shows the compound names, CAS numbers, chemical formulas, and retention index values, with the total number of essential oil samples. The essential oils of *T. quinquecostatus* populations mainly contained oxygenated monoterpenes, followed by monoterpene hydrocarbons. Six chemotypes, carvacrol, geraniol, geranyl acetate, linalool, *o*-cymene, and thymol, were distinguished according to the essential oil compositions (Table 3 and Figure 1). In particular, linalool (80.33%) was the most abundant component in sample T9. The maximum number of samples were grouped under the thymol chemotype (39), followed by the geraniol (30) and geranyl acetate (26) chemotypes.

### 2.3. Principal Component Analysis

The principal component analysis of 30 essential oil components in 103 *T. quinquecostatus* populations indicated that the first three principal components accounted for 90.014% of the total variation (Table 4). PC1 explained 65.916% of the total variance among the samples; additionally, PC2, PC3, and PC4 accounted for 13.482, 10.616, and 3.917% of the total variance, respectively. The major component PC1 exhibited a strong positive correlation with thymol (0.922), γ-terpinene (0.727), and β-pinene (0.705) components. However, it indicated a high negative correlation with geraniol (−0.922) and geranyl acetate (−0.842) components. In the case of PC2, a high correlation was observed with the concentrations of citral (0.766), nerol (0.757), and β-citral (0.754). Furthermore, PC3 showed a strong correlation with linalool (0.934).

**Figure 2:**
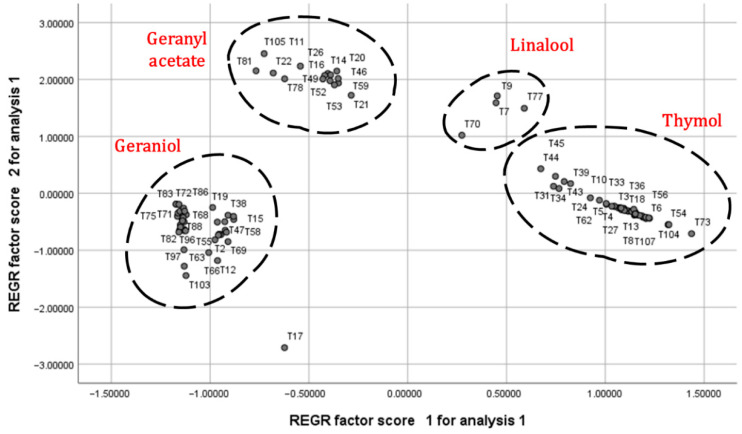
Scatter plot showing the similarity relationships among 103 individuals of *T. quinquecostatus* essential oils.

The scatter plot that was obtained from the scores of the principal components exhibited four distinct groups, and described 93.93% of the detected difference in the compositions of essential oils of *T. quinquecostatus* (Figure 2). Thymol, geraniol, geranyl acetate, and linalool (T7, T9, T70, and T77 samples) were the most significant components that were detected in the discrimination.

The principal components were found to be correlated with other chemical components. Table 5 shows the results of correlation coefficients among 30 essential oil components from *T. quinquecostatus* populations. In these, the essential oil components that exhibited significance at the 1% probability level, and had a correlation coefficient of 0.70 or higher, are presented here. The results indicated that citral (C19) showed a strong correlation with β-citral (0.957 **) and nerol (0.868 **). Nerol exhibited a strong correlation with β-citral (0.929 **), 3-thujene with α-pinene (0.721 **), and γ-terpinene (0.708 **), terpinolene (0.702 **), α-pinene with terpinen-4-ol (0.760 **), and α-terpineol (0.715 **). In addition, thujane-4-ol exhibited a high correlation with γ-terpinene (0.769 **), terpinolene with terpinen-4-ol (0.720 **), and terpinen-4-ol with α-terpineol (0.765 **). On the other hand, geraniol was negatively correlated with thymol (−0.839 ^**^) and β-pinene (−0.703 **).

**Figure 3:**
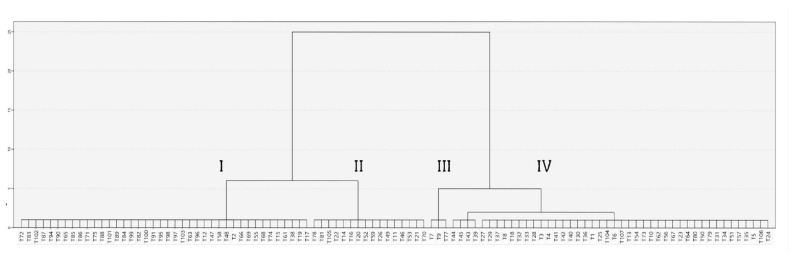
Dendrogram obtained by cluster analysis according to the chemical components of essential oils in 103 Korean native *T. quinquecostatus* individuals.

A dendrogram was constructed on the basis of of cluster analysis results (Figure 3). The essential oils from 103 *T. quinquecostatus* populations comprised 4 clusters. Group I contains individuals of *T. quinquecostatus* essential oils with the highest amount of geraniol. Group II comprises *T. quinquecostatus* individuals with the highest amount of geranyl acetate; Group III contains *T. quinquecostatus* individuals with the highest amount of linalool, and Group IV contains *T. quinquecostatus* individuals with the highest amount of thymol.

## 3. Discussion

*T. quinquecostatus* is a medicinally important aromatic plant in Korea. Traditionally, the essential oil of *T. quinquecostatus* has been used to treat various disorders, in addition to being used as a flavoring agent. The biological properties of the essential oils were highly correlated with the chemical class of the components. The types of essential oil components and their concentrations offer unique features to each essential oil. Furthermore, the most significant characteristic of an essential oil is its fragrance. Therefore, this study aimed to evaluate the chemical diversity within Korean native populations of *T. quinquecostatus*. For this purpose, seedlings of *T. quinquecostatus* collected from 103 different regions of Korea were grown under identical field conditions. The cultivated plants were harvested during the flowering stage, and the essential oils were isolated using the steam distillation technique. Kim et al. [5] also reported that the yield of essential oils from *T. quinquecostatus* ranged from 0.12 to 0.43%. In this study, 103 individuals of *T. quinquecostatus* were divided into six chemotypes, such as carvacrol, geraniol, geranyl acetate, linalool, o-cymene, and thymol, based on the major components found in their essential oils. In our previous study, Wolchul and Odae cultivars of Korean native *T. quinquecostatus* showed different chemotypes according to their essential oil compositions, even though they were placed into the same cluster on the basis of RAPD analysis results [5]. Previously, Shin and Kim [9], and Kim et al. [5], found that thymol (41.70 and 30.54%), γ-terpinene (16.00 and 23.92%), and p-cymene (13.00 and 11.13%) were major compounds in the essential oils of *T. quinquecostatus* (Supplementary Table S2).

Our previous study reported that the predominant compounds in the essential oils of *T. quinquecostatus* that were obtained from Wolchul and Jiri cultivars were different from the Odae cultivar. Geraniol (42.94%) and geranyl acetate (26.49%) were the most abundant components in the Wolchul cultivar, and linalool (47.89%) and thymol (15.98%) were the major compounds found in the Jiri cultivar [5]. Similarly with our report, *T. quinquecostatus* essential oils that were obtained from Cheongwon and Shandong Yimeng in China exhibited different chemotypes, such as trans-geraniol and *p*-cymene, respectively [19]. Chiang et al. [28] found that there was a significant variation in the essential oil compositions of *T. quinquecostatus* that were collected from different places in Korea, such as the high mountains of Jeju Island, the mid-mountainous region of Jeju, Gapyeong, and Ulleungdo. He et al. [21] identified 103 essential oil components from *T. quinquecostatus* collected from four different regions in China, and reported that 1,8-cineole, linalool, terpinen-4-ol, γ-terpinene, borneol, β-bisabolene, and α-pinene were commonly found in the essential oils that were obtained from all four regions. In particular, thymol and carvacrol were observed to possess identical structures, and that the degree of their transformation to each other may have been influenced by differences in environmental conditions [4]. It was also reported that the enzyme, geraniol dehydrogenase, specifically converts geraniol into geranial, and nerol into neral [29].

These studies revealed that various ecological and physiological factors play a major role in the composition of essential oils obtained from plants. However, the essential oils were isolated from *T. quinquecostatus*, which were grown under identical environmental conditions with similar edaphic factors. Therefore, the genetic makeup of *T. quinquecostatus* populations may be associated with their essential oil compositions. Quan et al. [30] demonstrated that *T. quinquecostatus* wild populations registered higher levels of genetic and clonal variations between patches within species. Rustaiee et al. [31] found genetic diversity and variations in the essential oil compositions of six *Thymus* species. Previous studies suggested that genetic diversity at the intraspecific level plays a crucial role in the composition of essential oils and their concentrations [32,33,34]. In addition, Choi et al. [35] suggested that harvesting time highly influenced the quality of thyme essential oils, and revealed that the essential oil content was found to be higher during the flowering period. Ghasemi Pirbalouti et al. [36] suggested that hybridization and introgression within species can also influence variations in the composition of essential oils of different *Thymus* species.

PCA can be employed as a valuable tool to understand relationships among the data. Furthermore, PCA is a useful pattern recognition technique to classify and discriminate plant samples, specifically essential oil compositions [16]. In the chemical composition, some of the components were present in only one essential oil, while other components were present in all of the essential oils. In this study, among 30 essential oil components that were selected from 103 *T. quinquecostatus* populations, the first three principal components accounted for 90.014% of the total variation. Thus, those 30 essential oil components were subsequently selected for PCA analysis. The cluster analysis supported the discrimination of *T. quinquecostatus* populations that was achieved through the PCA. *Thymus* species from different places in Iran were categorized into three chemotypes, such as thymol, geraniol/linalool, and carvacrol, according to cluster analysis [4].

A study reported that 11 *Thymus* species were classified into 3 major groups, according to morphological traits using PCA and cluster analyses [37]. A recent study also used PCA and cluster analyses to investigate differences in the composition of essential oils from five *Thymus* species, such as *T. atticus, T. leucotrichus, T. striatus, T. zygioides,* and *T. perinicus* [6]. Satyal et al. [38] found 20 different chemotypes in the essential oil compositions of 85 *T. vulgaris* samples, based on cluster analysis. Among them, 39 samples were grouped under the thymol chemotype. In another study, thymol and carvacrol were major essential oil components that were found in four *T. vulgaris* varieties, but significant variations were observed in the concentrations of major components in essential oils that were extracted before versus after the flowering stage [39].

On the basis of the essential oil composition-based dendrogram, *T. quinquecostatus* samples were classified into four major clusters. The results of correlations clearly demonstrated a relationship among Korean native populations of *T. quinquecostatus* and their chemical components (Table 5). Essential oil composition is a major factor used to detect a chemical between and within species. In this study, the statistical analysis results indicated that essential oil showed chemical diversity within the species, even though the collected seedlings were grown under identical field conditions.

## 4. Materials and Methods

### 4.1. Collection and Cultivation of T. quinquecostatus Populations

Seedlings of *T. quinquecostatus* were purchased from 107 different regions in the Korean peninsula. The collection period was between 2019 and 2020. The seedlings of *T. quinquecostatus* were separately planted in two rows at 25 cm intervals in the same experimental field, which was a 1650 m^2^ area that was located at Chuncheon, Gangwan-do, Republic of Korea. The plants were cultivated during the flowering season, and essential oils were extracted. Four samples were found decayed, due to being frozen. Thus, a total of 103 samples were used for further experimentation.

### 4.2. Essential Oil Extraction

The essential oils from the aerial parts of 103 populations of *T. quinquecostatus* were separately extracted using a steam distillation extraction technique. The plant samples were steam distilled at the boiling condition for 90 min, using a steam distillation apparatus (EssenLab Plus, Hanil Lab Tech Co., Ltd., Yangju, Korea). The yield (%, *v*/*w*) of essential oil was calculated on the basis of the weight of fresh sample. After extraction, the moisture in the isolated essential oil was removed using anhydrous sodium sulfate. The purified essential oil was kept at 4 °C prior to GC-MS analysis.

### 4.3. Gas Chromatography-Mass Spectrometry (GC-MS) Analysis

For the identification and quantitative analysis of components in the essential oils, a Varian CP3800 gas chromatograph coupled with a Varian 1200 L mass detector (Varian, Palo Alto, CA, USA) were used. The GC-MS was performed using a VF-5MS (Agilent, Santa Clara, CA, USA) polydimethylsiloxane (30 m × 0.25 mm × 0.25 µm) capillary column. The GC oven temperature was programmed from 50 °C (for 5 min) to 250 °C (for 3 min), at a rate of 5 °C/min, then increased to 300 °C at a rate of 20 °C/min; the final temperature was maintained for 5 min. The injector and the ion source temperature were 250 °C and 280 °C, respectively. A volume of 1 µL of the sample was injected, using a split ratio of 1:20. The carrier gas used was helium at a constant flow rate of 1 mL/min. For the mass spectra analysis, the ionization voltage was set to 70 eV, and the mass range was set to 30–500 *m/z*. The essential oil components of *T. quinquecostatus* were identified by comparing the mass spectrum data from the National Institute of Standards and Technology (NIST, 3.0) library, and the retention indices (RI) relative to a homologous series of n-alkanes (C_8_–C_20_) that was reported in the literature [40].

### 4.4. Statistical Analysis

For statistical analysis, the chemical compositions of essential oils of 103 *T. quinquecostatus* populations were integrated and sorted according to their RI values. Subsequently, the common essential oil components that appeared in 40 or more *T. quinquecostatus* individuals were selected separately from the raw data. Statistical analysis was performed on the basis of the extracted data, and then cluster analysis was carried out. PCA was carried out to analyze multiple data on the concentration of components in the essential oils, and to determine associations between the essential oil components and the collection sites of the *T. quinquecostatus* seedlings. All statistical analyses were carried out using IBM SPSS ver. 26 (IBM Corp. Released 2016, Chicago, IL, USA).

## 5. Conclusions

The data of the present study demonstrated that a significant chemical variation was observed within Korean native *T. quinquecostatus* populations based on the collection sites of the seedlings. One hundred and three *T. quinquecostatus* populations were grouped into six chemotypes, such as carvacrol, geraniol, geranyl acetate, linalool, o-cymene, and thymol. According to the PCA and cluster analysis, *T. quinquecostatus* populations could be categorized into four groups, such as thymol, geraniol, geranyl acetate, and linalool. These results indicated that the chemical composition of essential oils and their major components are excellent biomarkers to help understand the intraspecific variations among aromatic species. Further studies, in connection with genetic analyses of *T. quinquecostatus* populations, are necessary, in order to confirm the observed variations within the species.

## Figures and Tables

**Table 1:** Sampling sites of *T. quinquecostatus* and their essential oil yield.

Code No.	Sample Collection Site	Yield (% *v*/*w*)	Color of Essential Oil
T1	298, Maecheon-ro, Gwangui-myeon, Gurye-gun, Jeollanam-do	0.2	Orange
T2	310-11, Seonhwang-ro, Miam-myeon, Yeongam-gun, Jeollanam-do	0.5	Dark yellow
T3	1679, Gyeonggang-ro, Yongpyeong-myeon, Pyeongchang-gun, Gangwon-do	0.4	Orange
T4	330, Gimhwa-ro, Gimhwa-eup, Cheorwon-gun, Gangwon-do	0.5	Orange
T5	114-5, Heungjeonggyegok-gil, Bongpyeong-myeon, Pyeongchang-gun, Gangwon-do	0.5	Orange
T6	105-19, Ssukgogae-ro, Namwon-si, Jeollabuk-do	0.6	Dark yellow
T7	105-19, Ssukgogae-ro, Namwon-si, Jeollabuk-do	0.1	Lemon
T8	105-19, Ssukgogae-ro, Namwon-si, Jeollabuk-do	0.5	Red
T9	105-19, Ssukgogae-ro, Namwon-si, Jeollabuk-do	0.1	Lemon
T10	105-19, Ssukgogae-ro, Namwon-si, Jeollabuk-do	0.3	Yellow
T11	92, Yangjae-daero, Gwacheon-si, Gyeonggi-do	0.2	Yellow
T12	87, Iryeong-ro 502beon-gil, Jangheung-myeon, Yangju-si, Gyeonggi-do	0.4	Lemon
T13	109-3, Ijin-ri, Bukpyeong-myeon, Haenam-gun, Jeollanam-do	0.2	Dark yellow
T14	256-74, Hosu-ro, Ilsandong-gu, Goyang-si, Gyeonggi-do	0.3	Dark yellow
T15	256-113, Hosu-ro, Ilsandong-gu, Goyang-si, Gyeonggi-do	0.5	Lemon
T16	92, Yangjae-daero, Gwacheon-si, Gyeonggi-do	0.2	Yellow
T17	6, Andong-gil, Gurim-myeon, Sunchang-gun, Jeollabuk-do	0.3	Lemon
T18	1201, Bukbu-ro, Bongyang-eup, Jecheon-si, Chungcheongbuk-do	0.7	Dark yellow
T19	15, Choil-ro 174beon-gil, Hanam-si, Gyeonggi-do	0.2	Dark yellow
T20	80, Seonghyeon-ro, Gwanak-gu, Seoul	0.1	Lemon
T21	Iryeong-ro, Jangheung-myeon, Yangju-si, Gyeonggi-do	0.3	Dark yellow
T22	Wondang-ro, Deogyang-gu, Goyang-si, Gyeonggi-do	0.3	Yellow
T23	3914, Cheongsong-ro, Bunam-myeon, Cheongsong-gun, Gyeongsangbuk-do	0.2	Dark yellow
T24	563, Yeonju-ro, Jumunjin-eup, Gangneung-si, Gangwon-do	0.4	Orange
T25	52, Choil-ro 105beon-gil, Hanam-si, Gyeonggi-do	0.4	Dark yellow
T26	Juam-dong, Gwacheon-si, Gyeonggi-do	0.4	Orange
T27	Anseong-si, Gyeonggi-do	0.5	Red
T28	83-18, Gallyeong-gil, Ulleung-eup, Ulleung-gun, Gyeongsangbuk-do	0.4	Orange
T29	83-18, Gallyeong-gil, Ulleung-eup, Ulleung-gun, Gyeongsangbuk-do	0.5	Orange
T30	Cheonbu 3-gil, Buk-myeon, Ulleung-gun, Gyeongsangbuk-do	0.3	Orange
T31	Cheonbu 3-gil, Buk-myeon, Ulleung-gun, Gyeongsangbuk-do	0.4	Orange
T32	Dodong-ri, Ulleung-eup, Ulleung-gun, Gyeongsangbuk-do	0.5	Red
T33	Dodong 1-gil, Ulleung-eup, Ulleung-gun, Gyeongsangbuk-do	0.4	Red
T34	128-15, Chusan-gil, Buk-myeon, Ulleung-gun, Gyeongsangbuk-do 40207	0.4	Red
T35	90, Taeha-ri, Seo-myeon, Ulleung-gun, Gyeongsangbuk-do	0.7	Dark yellow
T36	Ulleungsunhwan-ro, Buk-myeon, Ulleung-gun, Gyeongsangbuk-do	0.3	Orange
T37	Na-ri, Buk-myeon, Ulleung-gun, Gyeongsangbuk-do	0.3	Orange
T38	256-74, Hosu-ro, Ilsandong-gu, Goyang-si, Gyeonggi-do	0.4	Lemon
T39	415, Gwangneungsumogwon-ro, Soheul-eup, Pocheon-si, Gyeonggi-do	0.5	Yellow
T40	72, Sumogwon-gil, Jeju-si, Jeju-do	0.6	Yellow
T41	72, Sumogwon-gil, Jeju-si, Jeju-do	0.5	Yellow
T42	72, Sumogwon-gil, Jeju-si, Jeju-do	0.4	Orange
T43	72, Sumogwon-gil, Jeju-si, Jeju-do	0.3	Orange
T44	72, Sumogwon-gil, Jeju-si, Jeju-do	0.3	Orange
T45	Gyorae-ri, Jocheon-eup, Jeju-si, Jeju-do	0.2	Orange
T46	300, Hallim-ro, Hallim-eup, Jeju-si, Jeju-do	0.3	Dark yellow
T47	Misan-dong, Siheung-si, Gyeonggi-do	0.5	Lemon
T48	24, Hongeunjungang-ro 3-gil, Seodaemun-gu, Seoul	0.5	Lemon
T49	72, Magokjungang 1-ro, Gangseo-gu, Seoul	0.3	Orange
T50	11-1, Cheondeoksan-ro 409beon-gil, Namsa-myeon, Cheoin-gu, Yongin-si, Gyeonggi-do	0.2	Yellow
T51	277-1, Maeho-gil, Hyeonnam-myeon, Yangyang-gun, Gangwon-do	0.6	Dark yellow
T52	21-1, Seoin-ro 1222beon-gil, Biin-myeon, Seocheon-gun, Chungcheongnam-do	0.3	Dark yellow
T53	172-31, Jinsan 2-gil, Nam-myeon, Taean-gun, Chungcheongnam-do	0.4	Dark yellow
T54	399-6, Geumgyedong-ro, Gonggeun-myeon, Hoengseong-gun, Gangwon-do	0.3	Dark yellow
T55	248, Howon-ro, Naeseo-eup, Masanhoewon-gu, Changwon-si, Gyeongsangnam-do	0.4	Lemon
T56	47-1, Baegam-ri, Yeomchi-eup, Asan-si, Chungcheongnam-do	0.2	Orange
T57	77, Hyoseongmunhak-gil, Bongpyeong-myeon, Pyeongchang-gun, Gangwon-do	0.5	Yellow
T58	122-1, Yonggang-ri, Dong-eup, Uichang-gu, Changwon-si, Gyeongsangnam-do	0.3	Lemon
T59	616, Hagui-ro, Uiwang-si, Gyeonggi-do	0.3	Yellow
T60	1192, Anyangpangyo-ro, Bundang-gu, Seongnam-si, Gyeonggi-do	**Froze to death**	
T61	256-116, Hosu-ro, Ilsandong-gu, Goyang-si, Gyeonggi-do	0.3	Lemon
T62	6, Dongtanjangjicheon 1-gil, Hwaseong-si, Gyeonggi-do	0.3	Dark Yellow
T63	1622, Hoguk-ro, Deogyang-gu, Goyang-si, Gyeonggi-do	0.5	Lemon
T64	91, Yangjae-daero, Gwacheon-si, Gyeonggi-do	0.8	Yellow
T65	256-85, Hosu-ro, Ilsandong-gu, Goyang-si, Gyeonggi-do	0.4	Transparent yellow
T66	49-20, Heonilleung 1-gil, Seocho-gu, Seoul	0.4	Lemon
T67	115, Beolmal-ro, Bucheon-si, Gyeonggi-do	0.2	Orange
T68	175, Angol-gil, Jewon-myeon, Geumsan-gun, Chungcheongnam-do	0.4	Lemon
T69	279-39, Eumnae-ro, Geoje-myeon, Geoje-si, Gyeongsangnam-do	0.6	Lemon
T70	44, Dangseong-ro 364 beon-gil, Songsan-myeon, Hwaseong-si, Gyeonggi-do	0.3	Orange
T71	93-1, Juam-dong, Gwacheon-si, Gyeonggi-do	0.5	Pale yellow
T72	133, Wangsimni-ro, Seongdong-gu, Seoul	0.5	Pale yellow
T73	51, Chusa-ro, Gwacheon-si, Gyeonggi-do	0.7	Yellow
T74	256-98, Hosu-ro, Ilsandong-gu, Goyang-si, Gyeonggi-do	0.3	Pale yellow
T75	217, Beolmal-ro, Bucheon-si, Gyeonggi-do	0.5	Transparent yellow
T76	13, Jeongung-ro, Namsa-myeon, Cheoin-gu, Yongin-si, Gyeonggi-do	**Froze to death**	
T77	513, Yongcheon-ro, Geumseong-myeon, Geumsan-gun, Chungcheongnam-do	0.2	Lemon
T78	557, Jungang-ro, Seocho-gu, Seoul	0.1	Yellow
T79	48-44, Hwadong-ro 587beon-gil, Ildong-myeon, Pocheon-si, Gyeonggi-do	0.3	Orange
T80	21-1, Seoin-ro 1222beon-gil, Biin-myeon, Seocheon-gun, Chungcheongnam-do	0.5	Orange
T81	5, Jinjinae 3-gil, Jeungpyeong-eup, Jeungpyeong-gun, Chungcheongbuk-do	0.2	Lemon
T82	22, Mareukbyeokjin-gil, Seo-gu, Gwangju	0.4	Pale yellow
T83	214-1, Samsang-ri, Jangheung-myeon, Yangju-si, Gyeonggi-do	0.4	Transparent yellow
T84	1660, Hoguk-ro, Deogyang-gu, Goyang-si, Gyeonggi-do	0.5	Lemon
T85	75-15, Songtangoga-gil, Jinwi-myeon, Pyeongtaek-si, Gyeonggi-do	0.5	Transparent yellow
T86	1006, Sansu-ro, Toechon-myeon, Gwangju-si, Gyeonggi-do	0.5	Transparent yellow
T87	24-53, Yeyang-gil, Yeondong-myeon, Sejong-si	0.5	Pale yellow
T88	1622, Hoguk-ro, Deogyang-gu, Goyang-si, Gyeonggi-do	0.7	Pale yellow
T89	329, Jeokcheon-ro, Muju-eup, Muju-gun, Jeollabuk-do	0.6	Pale yellow
T90	11, Daewangpangyo-ro 1000beon-gil, Sujeong-gu, Seongnam-si, Gyeonggi-do	0.6	Pale yellow
T91	39, Garim-ro, Gwangmyeong-si, Gyeonggi-do	0.5	Pale yellow
T92	256-74, Hosu-ro, Ilsandong-gu, Goyang-si, Gyeonggi-do	**Froze to death**	
T93	44, Dangseong-ro 364beon-gil, Songsan-myeon, Hwaseong-si, Gyeonggi-do	**Froze to death**	
T94	2-10, Sinin-gil, Asan-si, Chungcheongnam-do	0.6	Pale yellow
T95	212-114, Hwangmu-ro 330beon-gil, Sindun-myeon, Icheon-si, Gyeonggi-do	0.6	Transparent yellow
T96	852, Gyeryong-ro, Jung-gu, Daejeon	0.5	Pale yellow
T97	256-116, Hosu-ro, Ilsandong-gu, Goyang-si, Gyeonggi-do	0.3	Pale yellow
T98	496, Daehong-ro, Namil-myeon, Geumsan-gun, Chungcheongnam-do	0.5	Pale yellow
T99	53, Gyeongsu-daero, Uiwang-si, Gyeonggi-do	0.5	Pale yellow
T100	1192, Anyangpangyo-ro, Bundang-gu, Seongnam-si, Gyeonggi-do	0.6	Pale yellow
T101	460, Sugaesinam-gil, Namji-eup, Changnyeong-gun, Gyeongsangnam-do	0.5	Pale yellow
T102	115, Beolmal-ro, Bucheon-si, Gyeonggi-do	0.5	Pale yellow
T103	34, Doil-gil, Dong-myeon, Chuncheon-si, Gangwon-do	0.3	Pale yellow
T104	34, Doil-gil, Dong-myeon, Chuncheon-si, Gangwon-do	0.2	Yellow
T105	34, Doil-gil, Dong-myeon, Chuncheon-si, Gangwon-do	0.3	Dark Yellow
T106	114-5, Heungjeonggyegok-gil, Bongpyeong-myeon, Pyeongchang-gun, Gangwon-do	0.5	Red
T107	90-3, Onui-dong, Chuncheon-si, Gangwon-do	0.4	Dark Yellow

**Table 2:** Characteristics of essential oil components selected for PCA analysis.

Code	Compound Name	CAS No.	Formula	RI	Count
C1	1-Octen-3-ol	3391-86-4	C_8_H_16_O	942	89
C2	L-β-Pinene	18172-67-3	C_10_H_16_	990	78
C3	*o*-Cymene	527-84-4	C_10_H_14_	1027	47
C4	Eucalyptol	470-82-6	C_10_H_18_O	1034	58
C5	3-Thujene	353313	C_10_H_16_	1057	43
C6	D-α-Pinene	7785-70-8	C_10_H_16_	1059	75
C7	γ-Terpinene	99-85-4	C_10_H_16_	1060	85
C8	Camphene	79-92-5	C_10_H_16_	1063	62
C9	1-Nonen-3-ol	21964-44-3	C_9_H_18_O	1075	43
C10	Terpinolene	586-62-9	C_10_H_16_	1080	54
C11	cis-Thujane-4-ol	15537-55-0	C_10_H_18_O	1093	74
C12	Linalool	78-70-6	C_10_H_18_O	1100	84
C13	Isoborneol	10385-78-1	C_10_H_18_O	1170	98
C14	Terpinen-4-ol	562-74-3	C_10_H_18_O	1178	82
C15	α-Terpineol	98-55-5	C_10_H_18_O	1190	64
C16	β-Citral	106-26-3	C_10_H_16_O	1228	40
C17	Nerol	106-25-2	C_10_H_18_O	1238	54
C18	Geraniol	106-24-1	C_10_H_18_O	1239	68
C19	Citral	5392-40-5	C_10_H_16_O	1254	68
C20	Thymol	89-83-8	C_10_H_14_O	1272	99
C21	Carvacrol	499-75-2	C_10_H_14_O	1279	44
C22	Geranyl acetate	105-87-3	C_12_H_20_O_2_	1375	52
C23	Caryophyllene	87-44-5	C_15_H_24_	1419	102
C24	Humulene	6753-98-6	C_15_H_24_	1458	100
C25	β-Cubebene	13744-15-5	C_15_H_24_	1485	86
C26	Elixene	490377	C_15_H_24_	1500	43
C27	Butylated hydroxytoluene	128-37-0	C_15_H_24_O	1505	74
C28	β-Bisabolene	495-61-4	C_15_H_24_	1510	93
C29	β-Sesquiphellandrene	20307-83-9	C_15_H_24_	1527	56
C30	Caryophyllene oxide	1139-30-6	C_15_H_24_O	1589	90

**Table 3:** Chemotype classification of *T. quinquecostatus* populations.

Major Compounds	Code No. of Samples	No. of Samples
Carvacrol	T39, T43, T44, and T45	4
Geraniol	T11, T14, T16, T19, T20, T21, T22, T26, T46, T49, T52, T53, T59, T70, T71, T72, T75, T78, T81, T83, T85, T86, T87, T88, T89, T90, T94, T101, T102, and T105	30
Geranyl acetate	T2, T12, T15, T17, T38, T47, T48, T55, T58, T61, T63, T65, T66, T68, T69, T74, T82, T84, T91, T95, T96, T97, T98, T99, T100, and T103	26
Linalool	T7, T9, and T77	3
*o*-Cymene	T33	1
Thymol	T1, T3, T4, T5, T6, T8, T10, T13, T18, T23, T24, T25, T27, T28, T29, T30, T31, T32, T34, T35, T36, T37, T40, T41, T42, T50, T51, T54, T56, T57, T62, T64, T67, T73, T79, T80, T104, T106, and T107	39

**Table 4:** Principal component scores of the essential oil components in *T. quinquecostatus* populations.

No.	Code	Compound Name	Principal Components			
			PC1	PC2	PC3	PC4
1	C1	1-Octen-3-ol	0.597	−0.033	−0.038	0.275
2	C2	L-β-Pinene	0.705	−0.191	−0.108	0.214
3	C3	o-Cymene	0.617	−0.140	−0.113	0.330
4	C4	Eucalyptol	−0.209	0.269	−0.166	−0.111
5	C5	3-Thujene	0.693	−0.133	−0.097	0.296
6	C6	D-α-Pinene	0.682	−0.178	−0.116	0.224
7	C7	γ-Terpinene	0.727	−0.137	−0.116	0.165
8	C8	Camphene	0.454	−0.169	−0.085	0.117
9	C9	1-Nonen-3-ol	−0.182	−0.270	0.105	−0.013
10	C10	Terpinolene	0.640	−0.142	−0.102	0.182
11	C11	cis-Thujane-4-ol	0.586	−0.164	−0.109	0.129
12	C12	Linalool	0.099	0.280	0.934	−0.169
13	C13	Isoborneol	0.233	−0.098	−0.143	0.099
14	C14	Terpinen-4-ol	0.627	−0.159	−0.089	0.258
15	C15	α-Terpineol	0.633	−0.090	−0.107	0.312
16	C16	β-Citral	−0.169	0.754	−0.263	−0.058
17	C17	Nerol	−0.268	0.757	−0.263	−0.059
18	C18	Geraniol	−0.922	0.285	−0.220	−0.103
19	C19	Citral	−0.177	0.766	−0.268	−0.054
20	C20	Thymol	0.922	−0.224	−0.196	−0.230
21	C21	Carvacrol	0.239	0.122	0.015	0.775
22	C22	Geranyl acetate	−0.842	−0.531	0.087	−0.016
23	C23	Caryophyllene	0.170	0.382	0.586	0.180
24	C24	Humulene	0.264	−0.088	−0.054	−0.199
25	C25	β-Cubebene	0.228	0.443	0.554	−0.064
26	C26	Elixene	0.248	−0.140	0.087	0.317
27	C27	Butylated hydroxytoluene	0.100	0.221	0.212	−0.217
28	C28	β-Bisabolene	−0.283	0.050	−0.018	−0.028
29	C29	β-Sesquiphellandrene	0.133	0.085	0.489	−0.249
30	C30	Caryophyllene oxide	0.248	0.444	−0.190	0.083
% Variance			65.916	13.482	10.616	3.917
Cumulative variance			65.916	79.397	90.014	93.931

**Table 5:** Correlation coefficients between 30 essential oil components from *T. quinquecostatus* populations.

	(1)	(2)	(3)	(4)	(5)	(6)	(7)	(8)	(9)	(10)	(11)	(12)	(13)	(14)	(15)	(16)	(17)	(18)	(19)	(20)	(21)	(22)	(23)	(24)	(25)	(26)	(27)	(28)	(29)	(30)
^(1)^C1	1	0.560 **	0.401 **	−0.119	0.476 **	0.349 **	0.476 **	0.199 *	−0.184	0.418 **	0.471 **	−0.042	0.051	0.308 **	0.385 **	−0.139	−0.187	−0.584 **	−0.161	0.501 **	0.387 **	−0.495 **	0.187	0.342 **	0.180	0.515 **	−0.003	−0.245 *	0.140	0.109
^(2)^C2		1	0.544 **	−0.281 **	0.650 **	0.616 **	0.616 **	0.335 **	0.037	0.587 **	0.550 **	−0.126	0.170	0.594 **	0.584 **	−0.272 **	−0.339 **	−0.703 **	−0.289 **	0.656 **	0.273 **	−0.509 **	0.067	0.214 *	−0.019	0.343 **	−0.030	−0.197 *	0.000	0.115
^(3)^C3			1	−0.206 *	0.598 **	0.672 **	0.343 **	0.457 **	−0.029	0.597 **	0.273 **	−0.094	0.180	0.647 **	0.625 **	−0.202 *	−0.255 **	−0.586 **	−0.215 *	0.544 **	0.126	−0.458 **	0.135	−0.021	−0.075	0.195 *	−0.187	−0.186	−0.166	0.328 **
^(4)^C4				1	−0.244 *	−0.167	−0.285 **	−0.088	0.104	−0.254 **	−0.221 *	−0.095	0.049	−0.262 **	−0.145	0.392 **	0.454**	0.301 **	0.367 **	−0.196 *	−0.101	0.024	0.028	−0.070	0.075	−0.147	−0.060	0.284 **	0.145	0.108
^(5)^C5					1	0.721 **	0.708 **	0.589 **	−0.210^*^	0.702 **	0.578 **	−0.113	0.142	0.645 **	0.652 **	−0.236 *	−0.304 **	−0.685 **	−0.255 **	0.599 **	0.256 **	−0.531 **	0.098	−0.098	−0.140	0.366 **	−0.094	−0.342 **	−0.186	0.207 *
^(6)^C6						1	0.607 **	0.672 **	−0.121	0.672 **	0.425 **	−0.135	0.340 **	0.760 **	0.715 **	−0.241 *	−0.296 **	−0.681 **	−0.262 **	0.608 **	0.101	−0.500 **	0.091	−0.010	−0.169	0.253 **	−0.140	−0.211 *	−0.129	0.274 **
^(7)^C7							1	0.292 **	−0.239 *	0.576 **	0.769 **	−0.110	0.031	0.474 **	0.443 **	−0.238 *	−0.307 **	−0.700 **	−0.252 *	0.672 **	0.305 **	−0.554 **	0.074	0.003	−0.065	0.424 **	0.012	−0.411 **	−0.161	−0.016
^(8)^C8								1	−0.129	0.487 **	0.211 *	−0.109	0.603 **	0.521 **	0.599 **	−0.202 *	−0.220 *	−0.466**	−0.209 *	0.428 **	0.044	−0.308 **	0.060	−0.070	−0.148	0.148	−0.122	−0.143	−0.144	0.283 **
^(9)^C9									1	−0.269 **	−0.141	0.007	0.018	−0.037	−0.016	−0.218 *	−0.126	0.070	−0.219 *	−0.122	−0.100	0.304 **	0.008	−0.014	−0.068	−0.024	−0.043	0.297 **	0.072	−0.067
^(10)^C10										1	0.532 **	−0.095	0.133	0.720 **	0.550 **	−0.221 *	−0.282 **	−0.619 **	−0.234 *	0.587 **	0.155	−0.477 **	0.112	−0.017	−0.142	0.331 **	−0.023	−0.317 **	−0.129	0.084
^(11)^C11											1	−0.111	−0.027	0.394**	0.432 **	−0.229 *	−0.272 **	−0.571 **	−0.241 *	0.570 **	0.294 **	−0.417 **	0.042	0.008	−0.009	0.599 **	0.051	−0.358 **	−0.128	−0.104
^(12)^C12												1	−0.172	−0.113	−0.119	−0.066	−0.069	−0.188	−0.069	−0.108	−0.051	−0.147	0.642 **	−0.025	0.671 **	0.016	0.295 **	−0.057	0.517 **	−0.064
^(13)^C13													1	0.312 **	0.324 **	−0.065	−0.034	−0.239 *	−0.046	0.237 *	0.121	−0.161	−0.089	0.165	−0.022	−0.021	−0.084	0.248 *	0.078	0.174
^(14)^C14														1	0.765 **	−0.275 **	−0.310 **	−0.628 **	−0.288 **	0.550 **	0.166	−0.464 **	0.055	−0.002	−0.113	0.186	−0.002	−0.098	−0.057	0.222 *
^(15)^C15															1	−0.147	−0.194 *	−0.622 **	−0.178	0.540 **	0.271 **	−0.503 **	0.036	−0.013	−0.033	0.264 **	−0.095	−0.163	−0.106	0.329 **
^(16)^C16																1	0.929 **	0.394 **	0.957 **	−0.281 **	−0.095	−0.266 **	0.036	−0.104	0.125	−0.195 *	−0.055	0.062	−0.088	0.338 **
^(17)^C17																	1	0.497 **	0.868 **	−0.369 **	−0.107	−0.191	0.045	−0.131	0.102	−0.191	−0.064	0.134	−0.086	0.332**
^(18)^C18																		1	0.405 **	−0.839 **	−0.255 **	.607 **	−0.191	−0.243 *	−0.200 *	−0.315 **	−0.057	0.258 **	−0.193	−0.090
^(19)^C19																			1	−0.291 **	−0.086	−0.266 **	0.089	−0.103	0.156	−0.203 *	0.121	0.110	−0.099	.417 **
^(20)^C20																				1	0.055	−0.670 **	−0.096	0.340 **	0.032	0.171	0.054	−0.281 **	0.068	0.112
^(21)^C21																					1	−0.275 **	0.131	0.080	0.177	0.31 5**	−0.035	−0.151	−0.059	−0.071
^(22)^C22																						1	−0.301 **	−0.178	−0.377 **	−0.131	−0.183	0.202 *	−0.114	−0.469 **
^(23)^C23																							1	−0.161	0.507 **	0.219 *	0.341 **	0.014	0.294 **	0.320 **
^(24)^C24																								1	0.178	−0.080	0.230 *	0.165	0.611 **	−0.049
^(25)^C25																									1	0.147	0.349 **	0.030	0.547 **	0.127
^(26)^C26																										1	−0.061	−0.298 **	−0.092	−0.149
^(27)^C27																											1	0.081	0.306 **	0.218 *
^(28)^C28																												1	0.427 **	0.141
^(29)^C29																													1	−0.030
^(30)^C30																														1

(1)C1: 1-octen-3-ol, (2)C2: L-β-pinene, (3)C3: *o*-cymene, (4)C4: eucalyptol, (5)C5: 3-thujene, (6)C6: D-α-pinene, (7)C7: γ-terpinene, (8)C8; camphene, (9)C9: 1-nonen-3-ol, (10)C10: terpinolene, (11)C11: cis-thujane-4-ol, (12)C12: linalool, (13)C13: isoborneol, (14)C14: terpinen-4-ol, (15)C15: α-terpineol, (16)C16: β-citral (17)C17: nerol, (18)C18: geraniol, (19)C19: citral, (20)C20: thymol, (21)C21: Carvacrol, (22)C22: Geranyl acetate, (23)C23: Caryophyllene, (24)C24: humulene, (25)C25: β-cubebene, (26)C26: elixene, (27)C27: butylated hydroxytoluene, (28)C28: β-bisabolene, (29)C29: β-sesquiphellandrene, (30)C30: caryophyllene oxide. * Significant at the 5% level of probability. ** Significant at the 1% level of probability.

## Data Availability

Not applicable.

## Supplementary Materials

The following supporting information can be downloaded at: https://www.mdpi.com/article/10.3390/molecules27217203/s1, Table S1. The area percent of 30 components in the essential oils obtained from 103 *T. quinquecostatus* individuals. Table S2. Comparison of major essential oil components (area %) of *T. quinquecostatus* from previous publications

## Sample Availability

Samples of the essential oils of *Thymus quinquecostatus* are available from the authors.
