# Supplementary material for: Variations in the Chemical Composition of Essential Oils in Native Populations of Korean Thyme, Thymus quinquecostatus Celak
Source: Molecules. 2022 Oct 24;27(21):7203. doi: 10.3390/molecules27217203 (PMC9658411; doi:10.3390/molecules27217203)
Supplement: Supplementary file 1 [file molecules-27-07203-s001.zip › molecules-1974226-supplementary.pdf]

Table S1. The area percent of 30 components in the essential oils obtained from 103 *T. quinquecostatus* individuals.

RI*	Compound Name	CAS Number	Formula	Sample Name													
				T1	T2	T3	T4	T5	T6	T7	T8	T9	T10	T11	T12	T13	T14
942	1-Octen-3-ol	3391-86-4	$C_8H_{16}O$	2.92	0.68	0.00	1.29	0.81	1.44	0.61	0.97	0.70	2.22	0.22	0.32	0.15	0.34
990	L-β-Pinene	18172-67-3	$C_{10}H_{16}$	0.11	0.00	0.47	0.76	0.85	0.70	0.01	0.18	0.00	0.91	0.02	0.07	0.07	0.00
1027	o-Cymene	527-84-4	$C_{10}H_{14}$	18.55	0.00	29.53	23.09	0.00	0.00	0.03	34.52	0.00	0.00	0.00	0.00	0.00	0.00
1034	Eucalyptol	470-82-6	$C_{10}H_{18}O$	0.00	0.16	0.00	0.00	0.00	0.00	0.00	0.00	0.03	0.00	0.02	0.14	0.17	0.06
1057	3-Thujene	353313	$C_{10}H_{16}$	0.00	0.00	0.00	1.41	0.00	0.82	0.00	0.76	0.00	0.00	0.00	0.00	0.52	0.00
1059	D-α-Pinene	7785-70-8	$C_{10}H_{16}$	0.27	0.03	2.28	0.96	0.68	0.46	0.00	0.60	0.00	0.68	0.00	0.02	0.37	0.00
1060	γ-Terpinene	99-85-4	$C_{10}H_{16}$	2.79	0.11	15.40	17.65	19.31	12.19	0.02	2.62	0.01	5.89	0.00	0.08	7.67	0.03
1063	Camphene	79-92-5	$C_{10}H_{16}$	0.29	0.09	0.00	1.12	0.00	0.43	0.00	0.77	0.00	1.14	0.00	0.13	0.34	0.00
1075	1-Nonen-3-ol	21964-44-3	$C_9H_{18}O$	0.00	0.13	0.03	0.00	0.04	0.18	0.11	0.17	0.02	0.00	0.00	0.07	0.08	0.00
1080	Terpinolene	586-62-9	$C_{10}H_{16}$	0.00	0.00	2.16	2.28	2.34	0.00	0.00	1.10	0.00	0.28	0.00	0.02	0.77	0.00
1093	cis-Thujane-4-ol	15537-55-0	$C_{10}H_{18}O$	1.10	0.38	0.29	2.16	3.43	0.26	0.00	0.46	0.00	0.00	0.00	0.44	1.38	0.03
1100	Linalool	78-70-6	$C_{10}H_{18}O$	0.73	0.33	0.00	0.62	0.29	0.00	76.62	0.00	80.33	0.00	0.17	0.41	1.39	0.43
1170	Isoborneol	10385-78-1	$C_{10}H_{18}O$	4.51	2.93	1.26	4.03	0.00	0.67	0.06	1.32	0.11	7.51	1.26	4.29	0.60	1.61
1178	Terpinen-4-ol	562-74-3	$C_{10}H_{18}O$	0.00	0.00	2.83	1.10	0.00	0.47	0.11	1.01	0.00	0.43	0.00	0.46	0.69	0.00
1190	α-Terpineol	98-55-5	$C_{10}H_{18}O$	0.20	0.16	0.29	0.14	0.00	0.09	0.03	0.00	0.00	0.12	0.00	0.06	0.31	0.00
1228	β-Citral	106-26-3	$C_{10}H_{16}O$	0.00	0.00	0.02	0.00	0.00	0.00	0.00	0.00	0.00	0.00	11.66	0.00	0.00	14.23
1238	Nerol	106-25-2	$C_{10}H_{18}O$	0.00	0.45	0.00	0.00	0.00	0.00	0.00	0.00	0.01	0.00	2.68	0.00	0.00	2.86
1239	Geraniol	106-24-1	$C_{10}H_{18}O$	0.00	37.68	0.00	0.00	0.00	0.33	1.27	0.00	0.40	0.00	53.55	30.99	0.00	45.41
1254	Citral	5392-40-5	$C_{10}H_{16}O$	0.12	0.00	0.04	0.00	0.00	0.03	0.04	0.00	0.00	0.00	16.87	1.24	0.00	19.87
1272	Thymol	89-83-8	$C_{10}H_{14}O$	45.74	1.74	31.06	34.00	36.47	52.25	1.99	42.64	0.00	36.72	1.39	0.26	51.56	1.60
1279	Carvacrol	499-75-2	$C_{10}H_{14}O$	0.85	0.00	0.00	0.00	0.00	0.00	0.00	0.00	0.00	9.98	0.00	0.00	1.30	0.00
1375	Geranyl acetate	105-87-3	$C_{12}H_{20}O_2$	0.00	47.83	0.00	0.00	0.00	0.04	1.16	0.00	0.00	0.00	0.97	52.83	0.00	0.89
1419	Caryophyllene	87-44-5	$C_{15}H_{24}$	0.93	2.18	3.21	4.37	1.73	1.75	8.97	1.96	9.06	1.65	3.18	2.48	0.37	3.08
1458	Humulene	6753-98-6	$C_{15}H_{24}$	2.06	0.24	0.09	0.00	0.05	0.06	0.27	0.08	0.25	3.86	0.09	0.27	0.73	0.08
1485	β-Cubebene	13744-15-5	$C_{15}H_{24}$	0.72	0.22	0.07	0.00	0.12	0.08	4.09	0.05	3.45	1.11	0.93	0.24	1.05	0.80
1500	Elixene	490377	$C_{15}H_{24}$	0.00	0.00	0.00	0.64	0.07	0.00	0.54	0.00	0.06	0.11	0.00	0.22	0.00	0.00
1505	Butylated hydroxytoluene	128-37-0	$C_{15}H_{24}O$	0.00	0.00	0.00	0.00	0.22	0.20	0.49	0.00	0.86	0.24	0.00	0.28	0.59	0.25
1510	β-Bisabolene	495-61-4	$C_{15}H_{24}$	2.33	3.24	1.35	0.00	1.08	1.94	1.71	2.54	1.89	2.60	2.47	3.07	0.00	2.68
1527	β-Sesquiphellandrene	20307-83-9	$C_{15}H_{24}$	0.04	0.07	0.00	0.00	0.01	0.02	0.25	0.00	0.28	0.10	0.02	0.04	0.00	0.03
1589	Caryophyllene oxide	1139-30-6	$C_{15}H_{24}O$	0.29	0.20	0.72	0.78	0.27	0.15	0.37	0.66	0.22	0.19	1.14	0.21	0.49	0.75

*RI: retention index

RI*	Compound Name	CAS Number	Formula	Sample Name													
				T15	T16	T17	T18	T19	T20	T21	T22	T23	T24	T25	T26	T27	T28
942	1-Octen-3-ol	3391-86-4	$C_8H_{16}O$	0.72	0.27	0.45	1.97	0.06	0.40	0.54	0.54	0.00	1.70	0.00	0.76	0.00	0.00
990	L-β-Pinene	18172-67-3	$C_{10}H_{16}$	0.00	0.02	0.12	0.86	0.07	0.03	0.00	0.04	0.43	0.64	1.17	0.03	0.51	2.65
1027	o-Cymene	527-84-4	$C_{10}H_{14}$	0.64	0.09	0.00	27.34	0.00	0.34	0.00	0.11	7.97	0.00	18.49	0.25	26.19	30.70
1034	Eucalyptol	470-82-6	$C_{10}H_{18}O$	0.41	0.08	0.20	0.02	0.00	0.21	0.32	0.14	0.04	0.00	0.02	0.36	0.00	0.00
1057	3-Thujene	353313	$C_{10}H_{16}$	0.00	0.01	0.00	1.66	0.00	0.00	0.00	0.00	0.00	1.26	1.06	0.00	1.56	1.03
1059	D-α-Pinene	7785-70-8	$C_{10}H_{16}$	0.64	0.00	0.13	0.85	0.00	0.00	0.12	0.00	0.00	0.66	1.21	0.11	1.31	0.75
1060	γ-Terpinene	99-85-4	$C_{10}H_{16}$	0.52	0.04	0.18	0.00	0.01	0.12	0.21	0.04	2.98	19.24	0.00	0.11	8.45	4.52
1063	Camphene	79-92-5	$C_{10}H_{16}$	1.12	0.02	0.28	0.73	0.00	0.06	0.28	0.00	0.34	0.63	1.89	0.15	2.04	0.00
1075	1-Nonen-3-ol	21964-44-3	$C_9H_{18}O$	0.17	0.00	0.08	0.00	0.00	0.00	0.14	0.00	0.07	0.00	0.00	0.00	0.00	0.33
1080	Terpinolene	586-62-9	$C_{10}H_{16}$	0.00	0.00	0.06	7.28	0.00	0.00	0.05	0.00	0.06	2.39	3.36	0.00	1.95	0.00
1093	cis-Thujane-4-ol	15537-55-0	$C_{10}H_{18}O$	0.27	0.00	0.18	0.00	0.08	0.05	0.77	0.00	0.50	2.96	0.00	0.00	1.27	0.23
1100	Linalool	78-70-6	$C_{10}H_{18}O$	0.37	0.59	0.55	0.20	0.28	0.29	0.56	0.55	3.09	0.26	0.00	0.49	0.00	0.00
1170	Isoborneol	10385-78-1	$C_{10}H_{18}O$	3.74	3.01	4.64	1.30	3.02	2.93	6.88	1.36	5.04	0.99	11.17	1.66	6.04	1.33
1178	Terpinen-4-ol	562-74-3	$C_{10}H_{18}O$	0.40	0.00	0.00	2.45	0.32	0.00	0.75	0.31	0.75	1.72	3.04	0.00	1.77	1.71
1190	α-Terpineol	98-55-5	$C_{10}H_{18}O$	0.14	0.08	0.06	0.27	0.07	0.05	0.12	0.00	0.12	0.19	0.32	0.08	0.29	0.47
1228	β-Citral	106-26-3	$C_{10}H_{16}O$	0.33	14.02	0.00	0.00	0.59	14.89	0.21	1.54	0.00	0.00	0.00	13.06	0.00	0.00
1238	Nerol	106-25-2	$C_{10}H_{18}O$	0.52	4.34	0.00	0.00	0.53	3.05	0.72	2.16	0.00	0.00	0.00	2.04	0.34	0.00
1239	Geraniol	106-24-1	$C_{10}H_{18}O$	37.98	44.74	0.00	0.00	44.14	43.39	43.03	61.83	0.00	0.00	0.00	46.75	0.00	0.00
1254	Citral	5392-40-5	$C_{10}H_{16}O$	0.54	18.63	0.00	0.00	0.94	23.16	1.38	0.00	0.00	0.00	0.02	16.86	0.00	0.00
1272	Thymol	89-83-8	$C_{10}H_{14}O$	0.95	1.50	2.53	38.96	0.57	1.48	1.87	0.00	47.85	30.74	45.00	1.78	36.19	34.74
1279	Carvacrol	499-75-2	$C_{10}H_{14}O$	0.00	0.00	0.00	3.47	0.00	0.00	26.96	0.00	1.51	2.89	0.00	0.00	0.00	3.06
1375	Geranyl acetate	105-87-3	$C_{12}H_{20}O_2$	40.93	0.73	67.08	0.00	40.22	0.00	0.00	0.66	0.00	0.00	0.00	0.92	0.00	0.00
1419	Caryophyllene	87-44-5	$C_{15}H_{24}$	2.93	2.50	2.87	2.96	0.16	1.72	4.61	3.15	0.00	2.85	0.69	2.48	2.47	2.52
1458	Humulene	6753-98-6	$C_{15}H_{24}$	0.33	0.08	0.31	0.08	0.37	0.05	0.44	0.09	1.20	0.07	0.02	0.22	0.14	0.11
1485	β-Cubebene	13744-15-5	$C_{15}H_{24}$	0.00	0.61	0.26	0.08	0.69	0.29	0.38	0.69	3.15	0.08	0.08	0.61	0.06	0.09
1500	Elixene	490377	$C_{15}H_{24}$	0.52	0.00	0.26	0.34	0.05	0.00	0.00	0.00	0.00	0.28	0.00	0.00	0.38	0.00
1505	Butylated hydroxytoluene	128-37-0	$C_{15}H_{24}O$	0.00	0.22	0.00	0.22	0.30	0.00	0.00	0.37	0.00	0.18	0.26	0.00	0.00	0.19
1510	β-Bisabolene	495-61-4	$C_{15}H_{24}$	2.64	1.51	4.26	0.73	5.64	1.19	6.84	2.26	2.93	0.72	1.68	2.63	2.01	3.38
1527	β-Sesquiphellandrene	20307-83-9	$C_{15}H_{24}$	0.05	0.01	0.05	0.00	0.13	0.00	0.06	0.00	0.07	0.00	0.02	0.04	0.03	0.00
1589	Caryophyllene oxide	1139-30-6	$C_{15}H_{24}O$	0.24	0.95	0.29	0.19	0.33	0.61	0.33	1.56	0.02	0.17	0.09	0.90	0.68	1.03

*RI: retention index

RI*	Compound Name	CAS Number	Formula	Sample Name													
				T29	T30	T31	T32	T33	T34	T35	T36	T37	T38	T39	T40	T41	T42
942	1-Octen-3-ol	3391-86-4	$C_8H_{16}O$	1.89	0.88	1.18	1.50	1.57	2.32	0.91	0.80	1.57	0.55	0.84	3.45	2.72	3.89
990	L-β-Pinene	18172-67-3	$C_{10}H_{16}$	0.78	0.46	0.85	0.47	0.65	0.62	1.21	1.05	0.62	0.16	0.36	1.08	1.24	1.26
1027	o-Cymene	527-84-4	$C_{10}H_{14}$	22.95	18.85	0.00	28.93	33.82	0.00	9.40	16.04	25.00	0.00	0.00	16.86	19.09	19.04
1034	Eucalyptol	470-82-6	$C_{10}H_{18}O$	0.00	0.43	0.00	0.00	0.00	0.00	0.00	0.00	0.00	0.23	0.00	0.00	0.00	0.00
1057	3-Thujene	353313	$C_{10}H_{16}$	1.04	1.63	2.38	1.57	1.89	1.18	1.19	1.03	0.78	0.00	1.96	1.02	1.66	1.78
1059	D-α-Pinene	7785-70-8	$C_{10}H_{16}$	0.92	1.60	1.19	0.97	1.11	0.77	0.63	0.95	0.57	0.21	0.55	0.44	0.77	0.75
1060	γ-Terpinene	99-85-4	$C_{10}H_{16}$	6.75	6.71	10.01	10.05	7.24	4.35	21.82	7.77	7.51	0.10	26.31	14.17	13.15	13.42
1063	Camphene	79-92-5	$C_{10}H_{16}$	1.63	2.73	1.00	0.00	1.32	1.09	0.00	1.82	0.76	0.45	0.56	0.26	0.00	0.40
1075	1-Nonen-3-ol	21964-44-3	$C_9H_{18}O$	0.00	0.00	0.00	0.00	0.00	0.00	0.02	0.00	0.06	0.12	0.00	0.00	0.00	0.00
1080	Terpinolene	586-62-9	$C_{10}H_{16}$	1.58	1.97	0.28	2.20	2.02	1.81	3.20	1.70	1.50	0.04	0.15	0.11	2.10	2.36
1093	cis-Thujane-4-ol	15537-55-0	$C_{10}H_{18}O$	1.32	0.72	1.19	0.68	0.65	0.15	5.84	1.40	1.42	0.62	1.72	3.77	3.66	3.45
1100	Linalool	78-70-6	$C_{10}H_{18}O$	1.47	1.17	0.00	0.00	0.00	0.00	0.10	1.27	0.00	0.52	0.55	0.84	0.54	0.88
1170	Isoborneol	10385-78-1	$C_{10}H_{18}O$	3.90	6.37	0.00	1.91	3.04	3.72	0.58	6.05	4.52	5.38	8.08	0.36	0.36	0.34
1178	Terpinen-4-ol	562-74-3	$C_{10}H_{18}O$	1.22	1.22	1.48	0.93	1.36	2.16	2.73	0.81	1.43	0.39	0.76	0.84	1.06	0.92
1190	α-Terpineol	98-55-5	$C_{10}H_{18}O$	0.33	0.44	0.22	0.23	0.27	0.38	0.33	0.45	0.38	0.08	0.13	0.19	0.26	0.22
1228	β-Citral	106-26-3	$C_{10}H_{16}O$	0.00	0.00	0.00	0.00	0.00	0.00	0.00	0.00	0.00	0.00	0.00	0.00	0.00	0.00
1238	Nerol	106-25-2	$C_{10}H_{18}O$	0.00	0.00	0.00	0.00	0.39	0.00	0.00	0.00	0.00	1.28	0.00	0.00	0.00	0.00
1239	Geraniol	106-24-1	$C_{10}H_{18}O$	0.00	0.00	0.00	0.00	0.00	0.00	0.00	0.00	0.53	39.43	0.00	0.00	0.00	0.43
1254	Citral	5392-40-5	$C_{10}H_{16}O$	0.00	0.00	0.00	0.00	0.07	0.00	0.00	0.00	0.00	0.65	0.00	0.00	0.00	0.00
1272	Thymol	89-83-8	$C_{10}H_{14}O$	38.10	41.08	21.11	32.41	28.85	24.28	39.68	42.40	42.00	0.47	19.96	41.61	36.44	37.79
1279	Carvacrol	499-75-2	$C_{10}H_{14}O$	2.93	0.00	1.82	3.03	2.84	2.24	2.45	3.71	0.00	0.00	27.90	2.91	1.86	2.67
1375	Geranyl acetate	105-87-3	$C_{12}H_{20}O_2$	0.00	0.00	0.00	0.00	0.00	0.00	0.00	0.00	0.00	40.46	0.00	0.00	0.00	0.00
1419	Caryophyllene	87-44-5	$C_{15}H_{24}$	3.61	3.27	2.75	3.37	1.76	2.02	2.17	3.41	1.69	2.42	1.53	2.81	4.28	2.00
1458	Humulene	6753-98-6	$C_{15}H_{24}$	0.13	0.12	0.12	0.02	0.08	0.07	0.08	0.13	0.00	0.28	0.05	0.21	0.32	0.15
1485	β-Cubebene	13744-15-5	$C_{15}H_{24}$	0.49	0.44	0.14	0.00	0.06	0.14	0.25	0.57	0.00	0.25	0.65	1.01	0.85	1.14
1500	Elixene	490377	$C_{15}H_{24}$	0.00	0.00	0.00	0.00	0.00	0.00	0.20	0.20	0.00	0.29	0.38	1.11	1.54	1.17
1505	Butylated hydroxytoluene	128-37-0	$C_{15}H_{24}O$	0.28	0.00	0.32	0.18	0.00	0.28	0.56	0.22	0.00	0.13	0.24	0.24	0.00	0.00
1510	β-Bisabolene	495-61-4	$C_{15}H_{24}$	0.00	0.80	3.79	3.30	2.61	2.81	1.94	0.44	2.55	3.00	0.10	0.00	0.00	0.00
1527	β-Sesquiphellandrene	20307-83-9	$C_{15}H_{24}$	0.00	0.00	0.06	0.05	0.00	0.11	0.03	0.00	0.00	0.04	0.00	0.00	0.00	0.00
1589	Caryophyllene oxide	1139-30-6	$C_{15}H_{24}O$	0.93	1.10	0.81	0.77	1.06	1.47	0.07	1.33	0.71	0.21	0.13	0.00	0.58	0.27

*RI: retention index

RI*	Compound Name	CAS Number	Formula	Sample Name													
				T43	T44	T45	T46	T47	T48	T49	T50	T51	T52	T53	T54	T55	T56
942	1-Octen-3-ol	3391-86-4	$C_8H_{16}O$	3.27	3.38	1.49	0.21	0.76	0.69	0.63	2.19	1.40	0.79	0.92	1.13	0.93	1.41
990	L-β-Pinene	18172-67-3	$C_{10}H_{16}$	0.85	0.91	1.12	0.00	0.17	0.16	0.04	1.24	1.22	0.00	0.00	0.34	0.17	0.83
1027	o-Cymene	527-84-4	$C_{10}H_{14}$	21.23	8.27	14.43	0.00	0.00	0.05	0.39	0.00	7.96	0.45	0.99	0.00	0.00	9.56
1034	Eucalyptol	470-82-6	$C_{10}H_{18}O$	0.01	0.00	0.00	0.08	0.30	0.22	0.43	0.00	0.00	0.61	0.52	0.00	0.42	1.00
1057	3-Thujene	353313	$C_{10}H_{16}$	1.18	0.52	1.28	0.00	0.05	0.02	0.00	0.00	1.61	0.10	0.20	0.00	0.00	0.51
1059	D-α-Pinene	7785-70-8	$C_{10}H_{16}$	0.64	0.28	0.52	0.06	0.29	0.22	0.08	1.08	0.87	0.26	0.27	0.09	0.24	0.31
1060	γ-Terpinene	99-85-4	$C_{10}H_{16}$	12.21	12.57	9.92	0.11	0.27	0.10	0.30	8.86	25.71	0.66	2.73	17.58	0.09	4.58
1063	Camphene	79-92-5	$C_{10}H_{16}$	0.66	0.26	0.00	0.12	0.51	0.41	0.00	0.00	0.76	0.00	0.39	0.00	0.00	0.00
1075	1-Nonen-3-ol	21964-44-3	$C_9H_{18}O$	0.00	0.00	0.00	0.00	0.17	0.14	0.00	0.00	0.00	0.00	0.00	0.00	0.17	0.00
1080	Terpinolene	586-62-9	$C_{10}H_{16}$	1.93	1.53	1.66	0.00	0.00	0.00	0.03	0.22	2.87	0.08	0.00	1.11	0.00	1.02
1093	cis-Thujane-4-ol	15537-55-0	$C_{10}H_{18}O$	1.88	2.56	2.85	0.00	0.44	0.69	0.00	0.95	2.86	0.10	0.26	1.30	0.59	1.08
1100	Linalool	78-70-6	$C_{10}H_{18}O$	1.53	1.12	0.18	0.13	0.52	0.45	0.42	0.53	0.03	0.48	0.30	0.00	0.46	0.93
1170	Isoborneol	10385-78-1	$C_{10}H_{18}O$	1.71	0.80	0.00	2.82	3.03	2.60	1.57	5.93	0.90	1.71	1.94	0.82	0.00	2.65
1178	Terpinen-4-ol	562-74-3	$C_{10}H_{18}O$	1.58	0.78	0.65	0.00	0.44	0.18	0.00	0.52	0.53	0.00	0.00	0.75	0.26	0.40
1190	α-Terpineol	98-55-5	$C_{10}H_{18}O$	0.30	0.26	0.26	0.00	0.15	0.12	0.15	0.14	0.13	0.12	0.14	0.10	0.15	0.00
1228	β-Citral	106-26-3	$C_{10}H_{16}O$	0.00	0.00	0.00	1.82	0.34	0.32	11.70	0.00	0.00	12.80	9.24	0.00	0.31	0.00
1238	Nerol	106-25-2	$C_{10}H_{18}O$	0.00	0.00	0.00	0.00	0.59	0.53	2.71	0.00	0.00	2.92	2.69	0.31	0.57	0.00
1239	Geraniol	106-24-1	$C_{10}H_{18}O$	0.00	0.83	0.00	45.32	37.52	37.87	48.01	0.41	0.00	44.79	45.16	0.00	36.90	1.04
1254	Citral	5392-40-5	$C_{10}H_{16}O$	0.00	0.11	0.01	17.11	0.54	0.47	14.91	0.00	0.00	15.89	12.25	0.23	0.53	0.23
1272	Thymol	89-83-8	$C_{10}H_{14}O$	15.23	10.64	13.98	3.30	1.61	2.17	2.93	45.54	40.45	1.64	1.73	57.56	0.55	53.04
1279	Carvacrol	499-75-2	$C_{10}H_{14}O$	23.33	42.42	37.35	2.44	0.00	0.00	0.00	1.49	3.43	0.27	7.48	2.32	0.00	0.82
1375	Geranyl acetate	105-87-3	$C_{12}H_{20}O_2$	0.00	0.00	0.00	0.00	44.59	46.07	1.48	0.00	0.00	1.48	1.33	0.00	46.10	0.00
1419	Caryophyllene	87-44-5	$C_{15}H_{24}$	5.72	2.06	3.46	5.13	2.25	1.89	2.35	0.92	2.89	2.20	2.05	3.19	2.28	2.25
1458	Humulene	6753-98-6	$C_{15}H_{24}$	0.26	0.21	0.15	0.19	0.31	0.27	0.18	3.70	0.15	0.13	0.09	0.10	0.31	1.60
1485	β-Cubebene	13744-15-5	$C_{15}H_{24}$	0.25	2.03	1.01	1.43	0.28	0.24	0.85	1.20	0.15	0.72	0.68	0.12	0.27	1.04
1500	Elixene	490377	$C_{15}H_{24}$	0.66	1.57	0.08	0.00	0.39	0.00	0.00	0.33	0.51	0.00	0.00	0.42	0.39	0.00
1505	Butylated hydroxytoluene	128-37-0	$C_{15}H_{24}O$	0.50	0.00	0.00	1.66	0.17	0.18	0.00	0.21	0.54	0.24	0.00	0.40	0.19	0.00
1510	β-Bisabolene	495-61-4	$C_{15}H_{24}$	0.00	0.00	0.00	3.90	2.51	2.26	2.35	2.44	0.89	1.89	1.95	0.71	2.67	4.13
1527	β-Sesquiphellandrene	20307-83-9	$C_{15}H_{24}$	0.00	0.00	0.00	0.00	0.06	0.05	0.00	0.13	0.00	0.04	0.03	0.00	0.06	0.23
1589	Caryophyllene oxide	1139-30-6	$C_{15}H_{24}O$	0.39	0.16	0.00	1.95	0.14	0.26	0.62	0.00	0.07	0.55	0.41	0.15	0.27	0.46

*RI: retention index

RI*	Compound Name	CAS Number	Formula	Sample Name													
				T57	T58	T59	T61	T62	T63	T64	T65	T66	T67	T68	T69	T70	T71
942	1-Octen-3-ol	3391-86-4	$C_8H_{16}O$	1.91	0.82	0.74	0.79	1.81	0.35	0.51	0.00	0.62	2.47	0.52	0.68	0.82	0.23
990	L-β-Pinene	18172-67-3	$C_{10}H_{16}$	1.44	0.33	0.07	0.20	1.11	0.00	0.29	0.00	0.17	0.55	0.14	0.28	0.00	0.03
1027	o-Cymene	527-84-4	$C_{10}H_{14}$	8.96	0.17	1.25	0.27	4.77	0.00	7.51	0.00	0.00	8.17	0.00	0.00	2.71	0.00
1034	Eucalyptol	470-82-6	$C_{10}H_{18}O$	0.00	0.21	0.47	0.23	0.00	0.18	0.00	0.04	0.24	0.00	0.27	0.34	0.38	0.04
1057	3-Thujene	353313	$C_{10}H_{16}$	1.56	0.00	0.12	0.00	0.72	0.00	1.73	0.00	0.00	0.30	0.00	0.18	0.00	0.00
1059	D-α-Pinene	7785-70-8	$C_{10}H_{16}$	0.84	0.00	0.18	0.13	0.31	0.13	1.21	0.00	0.00	0.19	0.21	0.41	0.00	0.00
1060	γ-Terpinene	99-85-4	$C_{10}H_{16}$	22.23	0.28	0.44	0.69	4.72	0.04	15.25	0.00	0.11	2.38	0.06	0.43	6.23	0.02
1063	Camphene	79-92-5	$C_{10}H_{16}$	0.78	0.00	0.00	0.00	0.00	0.26	1.38	0.00	0.23	0.00	0.39	0.00	0.23	0.00
1075	1-Nonen-3-ol	21964-44-3	$C_9H_{18}O$	0.00	0.15	0.00	0.10	0.00	0.00	0.00	0.04	0.15	0.15	0.13	0.14	0.00	0.04
1080	Terpinolene	586-62-9	$C_{10}H_{16}$	3.24	0.05	0.00	0.06	1.10	0.00	2.94	0.00	0.00	0.55	0.00	0.12	0.53	0.00
1093	cis-Thujane-4-ol	15537-55-0	$C_{10}H_{18}O$	3.01	0.42	0.09	0.51	0.98	0.00	5.05	0.04	0.43	1.38	0.61	0.67	0.54	0.07
1100	Linalool	78-70-6	$C_{10}H_{18}O$	0.07	0.63	0.42	0.59	1.93	0.07	0.00	0.10	0.58	0.47	0.47	0.52	1.07	0.08
1170	Isoborneol	10385-78-1	$C_{10}H_{18}O$	1.82	2.70	1.52	2.69	0.81	1.36	3.77	1.36	2.85	2.93	2.50	2.86	1.50	0.96
1178	Terpinen-4-ol	562-74-3	$C_{10}H_{18}O$	0.00	0.30	0.00	0.21	0.34	0.07	1.16	0.00	0.25	0.56	0.17	0.20	0.25	0.04
1190	α-Terpineol	98-55-5	$C_{10}H_{18}O$	0.00	0.00	0.00	0.00	0.10	0.00	0.37	0.00	0.07	0.00	0.00	0.00	0.00	0.00
1228	β-Citral	106-26-3	$C_{10}H_{16}O$	0.00	0.31	12.29	0.41	0.00	0.00	0.00	0.00	0.24	0.00	0.29	0.31	4.19	0.08
1238	Nerol	106-25-2	$C_{10}H_{18}O$	0.14	0.54	2.56	0.55	0.00	0.00	0.00	0.11	0.55	0.00	0.52	0.47	1.35	0.12
1239	Geraniol	106-24-1	$C_{10}H_{18}O$	0.00	37.42	44.86	38.51	0.68	35.98	0.00	46.49	36.78	1.16	39.99	34.48	31.08	49.16
1254	Citral	5392-40-5	$C_{10}H_{16}O$	0.00	0.44	15.67	0.60	0.17	0.35	0.00	0.23	0.46	0.25	0.51	0.48	7.98	0.20
1272	Thymol	89-83-8	$C_{10}H_{14}O$	37.47	2.27	2.71	0.91	47.53	1.76	45.97	0.05	0.44	52.70	0.50	1.64	25.96	0.43
1279	Carvacrol	499-75-2	$C_{10}H_{14}O$	2.74	0.00	0.55	0.28	12.77	0.23	2.60	0.00	0.07	1.50	0.11	0.00	1.50	0.00
1375	Geranyl acetate	105-87-3	$C_{12}H_{20}O_2$	0.00	44.98	0.00	40.29	0.00	51.66	0.00	46.56	46.44	0.00	43.31	47.06	0.00	45.22
1419	Caryophyllene	87-44-5	$C_{15}H_{24}$	4.16	2.31	2.19	2.53	0.24	2.24	1.36	1.78	2.78	2.34	2.79	2.11	2.54	1.24
1458	Humulene	6753-98-6	$C_{15}H_{24}$	0.02	0.30	0.13	0.30	7.17	0.24	0.07	0.14	0.38	5.69	0.38	0.25	0.10	0.11
1485	β-Cubebene	13744-15-5	$C_{15}H_{24}$	0.00	0.26	0.72	0.31	0.00	0.19	0.09	0.08	0.28	1.34	0.31	0.23	0.91	0.07
1500	Elixene	490377	$C_{15}H_{24}$	0.83	0.36	0.00	0.00	0.00	0.02	1.46	0.21	0.42	0.00	0.44	0.33	0.00	0.16
1505	Butylated hydroxytoluene	128-37-0	$C_{15}H_{24}O$	0.00	0.25	0.29	0.25	0.40	0.49	0.29	0.14	0.25	0.59	0.27	0.31	0.79	0.14
1510	β-Bisabolene	495-61-4	$C_{15}H_{24}$	1.40	2.70	2.11	2.80	0.40	2.21	1.08	1.71	3.28	0.23	3.16	2.43	1.03	1.18
1527	β-Sesquiphellandrene	20307-83-9	$C_{15}H_{24}$	0.00	0.00	0.03	0.06	0.15	0.04	0.02	0.02	0.07	0.15	0.00	0.05	0.00	0.00
1589	Caryophyllene oxide	1139-30-6	$C_{15}H_{24}O$	0.23	0.18	0.59	0.28	0.00	0.14	0.00	0.10	0.18	1.05	0.22	0.14	0.27	0.00

*RI: retention index

RI*I	Compound Name	CAS Number	Formula	Sample Name													
				T72	T73	T74	T75	T77	T78	T79	T80	T81	T82	T83	T84	T85	T86
942	1-Octen-3-ol	3391-86-4	$C_8H_{16}O$	0.00	0.21	0.73	0.23	0.44	0.35	2.01	2.10	0.46	0.00	0.13	0.03	0.13	0.31
990	L-β-Pinene	18172-67-3	$C_{10}H_{16}$	0.00	0.61	0.15	0.00	0.00	0.00	0.66	1.35	0.00	0.02	0.02	0.03	0.02	0.02
1027	o-Cymene	527-84-4	$C_{10}H_{14}$	0.00	0.00	0.00	0.00	0.00	0.18	4.63	8.22	0.00	0.00	0.00	0.00	0.00	0.00
1034	Eucalyptol	470-82-6	$C_{10}H_{18}O$	0.00	0.00	0.21	0.03	0.00	0.44	0.00	0.00	0.42	0.00	0.00	0.05	0.00	0.01
1057	3-Thujene	353313	$C_{10}H_{16}$	0.00	0.49	0.00	0.00	0.00	0.00	0.00	1.04	0.00	0.00	0.00	0.00	0.00	0.00
1059	D-α-Pinene	7785-70-8	$C_{10}H_{16}$	0.00	0.39	0.30	0.00	0.05	0.06	0.14	0.75	0.00	0.00	0.00	0.04	0.00	0.00
1060	γ-Terpinene	99-85-4	$C_{10}H_{16}$	0.00	10.32	0.12	0.00	0.84	0.24	7.77	11.84	0.16	0.00	0.00	0.03	0.00	0.00
1063	Camphene	79-92-5	$C_{10}H_{16}$	0.00	0.50	0.00	0.00	0.00	0.00	0.19	1.04	0.00	0.00	0.00	0.08	0.00	0.00
1075	1-Nonen-3-ol	21964-44-3	$C_9H_{18}O$	0.00	0.04	0.10	0.00	0.00	0.00	0.00	0.00	0.00	0.11	0.03	0.00	0.02	0.03
1080	Terpinolene	586-62-9	$C_{10}H_{16}$	0.00	1.18	0.00	0.00	0.18	0.00	1.16	2.11	0.02	0.00	0.00	0.00	0.00	0.00
1093	cis-Thujane-4-ol	15537-55-0	$C_{10}H_{18}O$	0.00	5.02	0.56	0.06	0.00	0.08	0.82	0.55	0.05	0.00	0.00	0.00	0.04	0.00
1100	Linalool	78-70-6	$C_{10}H_{18}O$	0.08	0.00	0.45	0.10	77.48	0.22	0.36	0.76	0.36	0.06	0.08	0.13	0.05	0.06
1170	Isoborneol	10385-78-1	$C_{10}H_{18}O$	1.02	2.65	2.58	0.91	0.25	0.77	2.87	4.82	1.12	1.02	0.89	1.01	0.77	0.86
1178	Terpinen-4-ol	562-74-3	$C_{10}H_{18}O$	0.05	0.40	0.19	0.05	0.09	0.00	0.91	0.45	0.00	0.06	0.05	0.06	0.04	0.04
1190	α-Terpineol	98-55-5	$C_{10}H_{18}O$	0.00	0.10	0.00	0.01	0.02	0.00	0.15	0.00	0.00	0.00	0.00	0.00	0.01	0.00
1228	β-Citral	106-26-3	$C_{10}H_{16}O$	0.07	0.00	0.28	0.07	0.00	5.17	0.00	0.00	4.53	0.08	0.07	0.00	0.00	0.07
1238	Nerol	106-25-2	$C_{10}H_{18}O$	0.00	0.00	0.55	0.12	0.32	1.78	0.00	0.00	1.89	0.14	0.09	0.11	0.10	0.13
1239	Geraniol	106-24-1	$C_{10}H_{18}O$	52.04	0.00	39.17	49.15	0.00	59.27	0.86	0.00	65.00	45.58	52.16	46.65	49.88	49.75
1254	Citral	5392-40-5	$C_{10}H_{16}O$	0.18	0.00	0.45	0.17	0.11	8.27	0.13	0.00	6.50	0.18	0.17	0.19	0.17	0.19
1272	Thymol	89-83-8	$C_{10}H_{14}O$	0.00	67.18	1.17	0.12	8.65	4.16	48.60	45.47	1.40	0.07	0.09	0.14	0.25	0.08
1279	Carvacrol	499-75-2	$C_{10}H_{14}O$	0.00	2.66	0.18	0.00	0.18	0.28	0.00	0.00	0.49	0.00	0.00	0.00	0.00	0.00
1375	Geranyl acetate	105-87-3	$C_{12}H_{20}O_2$	43.02	0.00	42.43	45.88	0.00	3.32	0.00	0.00	3.79	48.98	43.03	47.69	45.00	44.38
1419	Caryophyllene	87-44-5	$C_{15}H_{24}$	1.27	1.19	2.08	1.12	4.33	3.33	1.76	3.44	2.78	1.35	1.26	1.26	1.27	1.38
1458	Humulene	6753-98-6	$C_{15}H_{24}$	0.00	0.03	0.24	0.09	0.13	0.20	7.94	0.45	0.17	0.11	0.11	0.11	0.11	0.12
1485	β-Cubebene	13744-15-5	$C_{15}H_{24}$	0.07	0.06	0.27	0.00	1.75	1.38	0.94	0.00	1.05	0.00	0.00	0.00	0.08	0.00
1500	Elixene	490377	$C_{15}H_{24}$	0.00	0.17	0.37	0.01	0.00	0.00	0.00	0.00	0.00	0.18	0.02	0.00	0.17	0.00
1505	Butylated hydroxytoluene	128-37-0	$C_{15}H_{24}O$	0.14	0.43	0.26	0.14	0.46	0.40	0.71	0.00	0.39	0.13	0.14	0.14	0.14	0.16
1510	β-Bisabolene	495-61-4	$C_{15}H_{24}$	1.11	0.61	2.35	1.19	0.86	2.64	5.98	0.85	1.87	1.43	1.36	1.34	1.43	1.52
1527	β-Sesquiphellandrene	20307-83-9	$C_{15}H_{24}$	0.00	0.00	0.05	0.00	0.09	0.00	0.31	0.00	0.08	0.02	0.02	0.00	0.02	0.02
1589	Caryophyllene oxide	1139-30-6	$C_{15}H_{24}O$	0.00	0.00	0.12	0.04	0.00	0.49	0.15	0.30	0.46	0.07	0.04	0.04	0.05	0.06

*RI: retention index

RI*	Compound Name	CAS Number	Formula	Sample Name													
				T87	T88	T89	T90	T91	T94	T95	T96	T97	T98	T99	T100	T101	T102
942	1-Octen-3-ol	3391-86-4	$C_8H_{16}O$	0.00	0.28	0.26	0.00	0.26	0.25	0.23	0.25	0.00	0.22	0.00	0.00	0.00	0.25
990	L-β-Pinene	18172-67-3	$C_{10}H_{16}$	0.00	0.03	0.00	0.04	0.00	0.02	0.03	0.00	0.00	0.03	0.00	0.00	0.01	0.00
1027	o-Cymene	527-84-4	$C_{10}H_{14}$	0.00	0.00	0.00	0.00	0.00	0.00	0.00	0.00	0.00	0.00	0.00	0.00	0.00	0.00
1034	Eucalyptol	470-82-6	$C_{10}H_{18}O$	0.00	0.05	0.07	0.06	0.08	0.05	0.07	0.05	0.06	0.05	0.00	0.03	0.06	0.00
1057	3-Thujene	353313	$C_{10}H_{16}$	0.00	0.00	0.00	0.00	0.00	0.00	0.00	0.00	0.00	0.00	0.00	0.00	0.00	0.00
1059	D-α-Pinene	7785-70-8	$C_{10}H_{16}$	0.00	0.00	0.00	0.11	0.08	0.00	0.00	0.04	0.10	0.00	0.00	0.05	0.11	0.00
1060	γ-Terpinene	99-85-4	$C_{10}H_{16}$	0.00	0.00	0.03	0.02	0.03	0.02	0.00	0.03	0.05	0.00	0.00	0.00	0.02	0.00
1063	Camphene	79-92-5	$C_{10}H_{16}$	0.00	0.00	0.17	0.23	0.17	0.08	0.00	0.08	0.23	0.13	0.00	0.09	0.22	0.06
1075	1-Nonen-3-ol	21964-44-3	$C_9H_{18}O$	0.12	0.03	0.04	0.04	0.04	0.03	0.00	0.00	0.00	0.03	0.00	0.20	0.00	0.00
1080	Terpinolene	586-62-9	$C_{10}H_{16}$	0.00	0.00	0.00	0.00	0.00	0.00	0.00	0.00	0.00	0.00	0.00	0.00	0.00	0.00
1093	cis-Thujane-4-ol	15537-55-0	$C_{10}H_{18}O$	0.00	0.00	0.10	0.00	0.00	0.05	0.07	0.00	0.00	0.10	0.00	0.00	0.00	0.00
1100	Linalool	78-70-6	$C_{10}H_{18}O$	0.06	0.07	0.06	0.07	0.08	0.07	0.07	0.00	0.00	0.05	0.00	0.06	0.07	0.00
1170	Isoborneol	10385-78-1	$C_{10}H_{18}O$	0.94	1.00	1.11	1.16	1.15	1.06	0.97	1.08	0.00	1.13	0.98	0.89	1.48	0.92
1178	Terpinen-4-ol	562-74-3	$C_{10}H_{18}O$	0.05	0.00	0.06	0.04	0.05	0.05	0.06	0.05	0.05	0.04	0.04	0.04	0.04	0.06
1190	α-Terpineol	98-55-5	$C_{10}H_{18}O$	0.00	0.00	0.00	0.00	0.01	0.01	0.00	0.00	0.00	0.00	0.00	0.07	0.00	0.00
1228	β-Citral	106-26-3	$C_{10}H_{16}O$	0.07	0.08	0.09	0.10	0.09	0.00	0.00	0.00	0.00	0.00	0.00	0.00	0.00	0.00
1238	Nerol	106-25-2	$C_{10}H_{18}O$	0.10	0.11	0.14	0.14	0.12	0.11	0.11	0.09	0.00	0.00	0.13	0.10	0.11	0.00
1239	Geraniol	106-24-1	$C_{10}H_{18}O$	47.58	49.68	47.66	47.63	44.85	47.12	44.87	40.24	36.40	44.22	46.06	45.25	48.81	51.50
1254	Citral	5392-40-5	$C_{10}H_{16}O$	0.15	0.18	0.21	0.23	0.22	0.17	0.17	0.14	0.14	0.18	0.18	0.16	0.16	0.16
1272	Thymol	89-83-8	$C_{10}H_{14}O$	0.38	0.06	0.08	0.04	0.10	0.12	0.43	0.06	0.00	0.10	0.07	0.07	0.06	0.06
1279	Carvacrol	499-75-2	$C_{10}H_{14}O$	0.00	0.00	0.00	0.00	0.00	0.00	0.00	0.00	0.00	0.00	0.00	0.00	0.00	0.00
1375	Geranyl acetate	105-87-3	$C_{12}H_{20}O_2$	46.26	43.22	44.56	45.59	48.41	46.45	48.74	53.42	57.43	48.57	48.21	49.61	43.84	42.75
1419	Caryophyllene	87-44-5	$C_{15}H_{24}$	1.62	1.83	2.02	1.57	1.62	1.63	1.56	1.52	1.63	1.88	1.66	1.28	1.67	1.50
1458	Humulene	6753-98-6	$C_{15}H_{24}$	0.13	0.15	0.16	0.12	0.12	0.13	0.13	0.10	0.12	0.15	0.14	0.10	0.11	0.11
1485	β-Cubebene	13744-15-5	$C_{15}H_{24}$	0.00	0.10	0.10	0.07	0.00	0.00	0.00	0.07	0.08	0.09	0.00	0.06	0.07	0.08
1500	Elixene	490377	$C_{15}H_{24}$	0.00	0.00	0.00	0.00	0.00	0.19	0.00	0.00	0.00	0.00	0.00	0.00	0.00	0.00
1505	Butylated hydroxytoluene	128-37-0	$C_{15}H_{24}O$	0.12	0.16	0.00	0.15	0.16	0.13	0.00	0.13	0.00	0.16	0.16	0.16	0.07	0.15
1510	β-Bisabolene	495-61-4	$C_{15}H_{24}$	1.74	2.12	2.34	1.62	1.61	1.64	1.85	1.86	1.92	1.94	1.69	1.36	1.78	1.66
1527	β-Sesquiphellandrene	20307-83-9	$C_{15}H_{24}$	0.02	0.03	0.03	0.00	0.00	0.02	0.00	0.00	0.00	0.02	0.00	0.00	0.01	0.00
1589	Caryophyllene oxide	1139-30-6	$C_{15}H_{24}O$	0.05	0.05	0.07	0.07	0.00	0.04	0.05	0.09	0.07	0.00	0.06	0.00	0.05	0.00

*RI: retention index

RI*	Compound Name	CAS Number	Formula	Sample Name				
				T103	T104	T105	T106	T107
942	1-Octen-3-ol	3391-86-4	$C_8H_{16}O$	0.14	0.85	0.49	0.64	1.12
990	L-β-Pinene	18172-67-3	$C_{10}H_{16}$	0.04	0.17	0.00	0.88	0.22
1027	o-Cymene	527-84-4	$C_{10}H_{14}$	0.00	22.24	0.10	0.00	0.00
1034	Eucalyptol	470-82-6	$C_{10}H_{18}O$	0.08	0.27	0.14	0.00	0.23
1057	3-Thujene	353313	$C_{10}H_{16}$	0.00	1.08	0.00	1.81	1.23
1059	D-α-Pinene	7785-70-8	$C_{10}H_{16}$	0.04	0.48	0.01	0.74	0.53
1060	γ-Terpinene	99-85-4	$C_{10}H_{16}$	0.00	11.14	0.06	20.53	14.67
1063	Camphene	79-92-5	$C_{10}H_{16}$	0.19	0.22	0.03	0.51	0.23
1075	1-Nonen-3-ol	21964-44-3	$C_9H_{18}O$	0.00	0.00	0.00	0.04	0.00
1080	Terpinolene	586-62-9	$C_{10}H_{16}$	0.03	1.07	0.00	2.70	0.05
1093	cis-Thujane-4-ol	15537-55-0	$C_{10}H_{18}O$	0.00	0.78	0.06	2.43	0.89
1100	Linalool	78-70-6	$C_{10}H_{18}O$	0.06	1.40	0.24	0.17	1.36
1170	Isoborneol	10385-78-1	$C_{10}H_{18}O$	1.64	0.58	1.16	1.49	0.55
1178	Terpinen-4-ol	562-74-3	$C_{10}H_{18}O$	0.04	0.38	0.00	1.35	0.38
1190	α-Terpineol	98-55-5	$C_{10}H_{18}O$	0.02	0.15	0.10	0.19	0.17
1228	β-Citral	106-26-3	$C_{10}H_{16}O$	0.00	0.00	8.62	0.00	0.11
1238	Nerol	106-25-2	$C_{10}H_{18}O$	0.08	0.00	2.42	0.00	0.00
1239	Geraniol	106-24-1	$C_{10}H_{18}O$	34.08	0.15	65.28	0.00	0.51
1254	Citral	5392-40-5	$C_{10}H_{16}O$	0.17	0.00	10.67	0.00	0.16
1272	Thymol	89-83-8	$C_{10}H_{14}O$	0.14	52.95	0.93	37.89	52.62
1279	Carvacrol	499-75-2	$C_{10}H_{14}O$	0.00	0.00	0.00	0.00	0.00
1375	Geranyl acetate	105-87-3	$C_{12}H_{20}O_2$	59.57	0.00	0.00	0.00	0.00
1419	Caryophyllene	87-44-5	$C_{15}H_{24}$	0.85	1.47	1.98	1.03	1.40
1458	Humulene	6753-98-6	$C_{15}H_{24}$	0.09	0.04	0.07	0.04	0.04
1485	β-Cubebene	13744-15-5	$C_{15}H_{24}$	0.08	0.19	0.33	0.09	0.19
1500	Elixene	490377	$C_{15}H_{24}$	0.00	0.00	0.00	0.00	0.00
1505	Butylated hydroxytoluene	128-37-0	$C_{15}H_{24}O$	0.03	0.07	0.00	0.04	0.06
1510	β-Bisabolene	495-61-4	$C_{15}H_{24}$	1.29	0.00	1.26	0.57	0.07
1527	β-Sesquiphellandrene	20307-83-9	$C_{15}H_{24}$	0.02	0.00	0.00	0.02	0.00
1589	Caryophyllene oxide	1139-30-6	$C_{15}H_{24}O$	0.18	0.36	0.52	0.09	0.22

*RI: retention index

Table S2. Comparison of major essential oil components (area %) of *T. quinquecostatus* from previous publications

Compounds	Jeju Island, Korea [20]	Korea [22]	Seoul, Korea [9]	YL, Shaanxi, China [21]	JB, Shaanxi, China [21]	QY, Gansu, China [21]	Ningxia Hui, China [21]	Odae Mt, Korea [5]	Wolchul Mt, Korea [5]	Jiri Mt, Korea [5]	Mt. Laoshan, Qingdao, China [23]		
											Growth period	Flowering period	Withered period
1,8-Cineole	-	-	-	7.23	5.22	10.16	1.84	-	-	-	-	-	-
1-4-Terpineneol	-	-	-	-	-	-	-	-	-	-	1.86	2.14	2.56
1-Octen-3-ol	-	-	-	-	-	-	-	-	-	3.29	-	-	-
Borneol	-	-	-	6.50	5.61	-	1.38	2.81	5.91	-	-	14.58	-
Camphene	0.62	4.40	-	-	-	-	-	-	-	-	-	-	-
Camphor	-	5.90	-	-	-	-	-	-	-	-	-	-	-
Carvacrol	-	2.60	4.00	-	-	-	-	-	-	-	-	-	-
Carvacrol methyl ether	-	-	-	31.80	23.32	-	-	-	-	-	-	-	-
Caryophyllene	-	-	4.70	-	-	-	-	4.74	4.73	7.02	4.51	3.82	2.44
Cymene	19.04	-	-	-	-	-	-	-	-	-	-	-	-
Geraniol	-	-	-	-	-	-	-	-	42.94	-	-	-	-
Geranyl acetate	-	-	-	-	-	-	-	-	26.49	-	-	-	-
Linalool	-	-	-	-	-	-	12.80	-	-	47.89	40.31	39.10	45.44
Neral	-	-	-	-	-	-	1.35	-	-	-	-	-	-
o-Cymene	-	-	-	-	-	12.10	-	-	-	-	-	-	-
p-Cyemn-2ol	20.06	-	-	-	-	-	-	-	-	-	-	-	-
p-Cyemn-3ol	50.41	-	-	-	-	-	-	-	-	-	-	-	-
p-Cymene	-	9.20	13.00	-	19.20	-	-	11.13	-	-	-	-	-
p-Vinyl guaiacol	-	-	-	3.97	-	23.55	-	-	-	-	-	-	-
Terpinen-4-ol	-	-	-	4.96	10.56	-	-	-	-	-	-	-	-
Terpinolene	-	-	-	-	-	-	-	3.55	-	-	-	-	-
Thymol	-	39.80	41.70	-	-	16.32	-	30.54	-	15.98	-	-	-
α-Elemol	-	-	-	-	-	-	-	-	-	-	3.54	3.03	1.87
α-Pinene	-	2.60	-	-	-	-	-	-	-	-	-	-	-
α-Terpineol	-	4.50	-	-	-	-	-	-	-	-	-	-	-

β-Bisabolene	-	3.30	-	-	-	-	-	-	3.86	3.00	-	-	-
β-Cadinene	-	-	-	-	-	-	-	-	-	-	5.57	4.13	3.18
β-Cubebene	-	-	-	-	-	-	-	-	-	3.47	-	-	-
β-Eudesmol	-	-	-	-	-	-	-	-	-	-	2.10	-	-
β-Linalool	1.73	-	-	-	-	-	-	-	-	-	-	-	-
γ-Terpinene	0.96	10.00	16.00	3.03	2.73	11.11	-	23.92	-	3.43	-	-	-
γ-Terpineol	-	-	-	-	-	-	3.04	-	-	-	-	-	-

Numbers in the parenthesis are Reference number in the main text.
